# Blood Flow Restriction Training Prior to and After Anterior Cruciate Ligament Reconstruction: A Scoping Review

**DOI:** 10.3390/jfmk10040450

**Published:** 2025-11-19

**Authors:** Roger Fontanet, Rafel Donat, Eduardo Carballeira

**Affiliations:** 1 Sport, Exercise and Human Movement (SEaHM), University of Vic—Central University of Catalonia (UVic-UCC), 08242 Manresa, Spain; rfontanet@umanresa.cat; 2 Educational Technology and Knowledge Management, Research Group Gribopofet, University of Vic—Central University of Catalonia (UVic-UCC), 08242 Manresa, Spain; rdonat@umanresa.cat; 3 Department of Specific Didactics, Physical Education and Sports Area, University of La Laguna (ULL), 38200 La Laguna, Spain

**Keywords:** anterior cruciate ligament reconstruction, blood flow restriction, preoperative rehabilitation, postoperative rehabilitation, exercise parameters

## Abstract

**Background**: Anterior cruciate ligament injuries often lead to muscle atrophy and prolonged recovery following anterior cruciate ligament reconstruction (ACLR). Blood flow restriction (BFR) has emerged as a strategy to optimize neuromuscular adaptations with lower loads, potentially enhancing rehabilitation outcomes in both preoperative and postoperative phases. This review aims to comprehensively evaluate the comparative effectiveness of BFR applied during resistance and endurance exercises versus non-BFR protocols, both before and after ACLR, while also examining key training parameters and BFR protocols to guide further research and clinical practice. **Methods**: A comprehensive literature search was conducted across multiple databases, including WoS, PEDro, Scopus, PUBMED (MEDLINE), SportDiscus, and the Cochrane Library, covering publications from inception to 24 September 2025. Studies eligible for inclusion were randomized controlled trials and quasi-randomized controlled trials that compared BFR interventions with non-BFR training in patients undergoing ACLR. Data synthesis followed the recommendations of the PRISMA Extension for Scoping Reviews (PRISMA-ScR). The PEDro and CERT scales were used to assess the methodological quality of the included studies. Detailed training parameters and cuff specifications were extracted and are summarized in tables. **Results**: In total, 15 of the initial 441 articles identified met the eligibility criteria and were included in the final analysis, comprising a sample of 417 patients. Outcomes were categorized into six areas: body composition, neuromuscular responses and adaptations, self-report questionnaires, functional measures, muscle physiology and biomarkers, and return to activity. Five articles focused on preoperative interventions, nine focused on postoperative interventions, and one addressed both phases. **Conclusions**: This review suggests that BFR resistance training is an effective tool in the preoperative and postoperative phases of ACLR. Additionally, it can help improve muscle size, strength, functional measurements, body composition, muscle blood flow, and subjective perceptions.

## 1. Introduction

The occurrence of anterior cruciate ligament (ACL) injuries has been increasing, notably affecting adolescent athletes [1,2,3] and the general population [4,5]. The annual incidence is estimated to be approximately 0.01% to 0.05% in the general population, 0.15 to 7.32% in professional athletes, and 0.002 to 1.62% in amateur athletes [6]. Over the past few decades, there has been a significant increase in ACL injuries among young athletes, with increases ranging from 44% to 143% [2,7]. ACL injury directly impacts the quality of life of injured individuals and has a high socioeconomic impact in both the short and long term [8,9].

In the early stages following ACL reconstruction (ACLR), postoperative factors such as immobilization, restricted weight bearing, and the use of an intraoperative tourniquet, together with the temporary inability to perform heavy load resistance training (HL-RT) and/or high-intensity endurance exercises, collectively contribute to muscular atrophy and weakness in the lower extremities [10,11]. This particularly affects the quadriceps, which is a clinical manifestation and primary risk factor for the development of knee osteoarthritis [12], and leads to deconditioning in the injured individual, thereby delaying their return to activity. Muscular strength and hypertrophy are primarily induced through increased firing rates and increased recruitment of motor neurons, thereby resulting in increased mechanical tension within engaged myofibers [13,14]. The optimal development of maximal strength and power is achieved by employing heavy loads (≥85% of one repetition maximum, 1RM) and moderate loads (40–70% 1RM), respectively [15]. However, traditional muscle-strengthening training involving near-maximal loads or engaging in contractions at maximum voluntary velocity is not recommended during the initial stages following ACLR [16]. Consequently, there has been a growing interest in blood flow restriction (BFR) applied to proximal limbs during exercise as a potentially effective method. This is primarily due to the fact that BFR training combines high local mechanical tension, hypoxia, and metabolic accumulation, which simultaneously potentiate hypertrophic and metabolic remodeling pathways. The reduction in tissue oxygen availability increases the recruitment of high-threshold motor units and electromyographic excitation, even under low external loads [17,18], thereby reproducing the neuromuscular effects of high-intensity exercise. This environment activates the mTOR–S6K1 signaling pathway, responsible for enhancing protein synthesis and muscle mass, while also stimulating angiogenic mediators such as VEGF, VEGFR-2, HIF-1α, and eNOS, together with mitochondrial biogenesis regulators (PGC-1α, AMPK) that promote capillarization and oxidative efficiency [19,20]. Moreover, the interaction between mTOR and PGC-1α, reported in studies of mitochondrial remodeling [21], suggests that the mechanical tension and hypoxia induced by BFR elicit an integrated adaptive response capable of improving muscular strength and metabolic capacity while requiring lower external loads.

Such adaptations could offer benefits in both the preoperative (PRE-OP) and postoperative (POST-OP) phases of ACLR while also providing a peripheral stimulus to improve cardiovascular fitness [17,20], which is often diminished after ACLR rehabilitation compared with preinjury values [22].

Blood flow restriction resistance training (BFR-RT) and BFR endurance training (BFR-ET) have emerged as promising alternatives to traditional HL-RT or high-intensity training [19,23]; these methods can elicit similar neuromuscular adaptations in certain populations and offer benefits such as alleviating knee pain, reducing swelling, and improving functionality [23,24,25]. Furthermore, BFR-RT has been implemented in PRE-OP phases to confer protective effects against ischemia-induced injury and bolster muscle strength and endurance for faster rehabilitation [26]. On the other hand, BFR-RT applied during the POST-OP phase enhances skeletal muscle hypertrophy and functional measurements to a similar extent as does HL-RT [27]. Furthermore, BFR-ET during the POST-OP phase resulted in reduced moments around the knee joint compared with the no-BFR condition, suggesting that BFR-ET is a safe option to utilize following ACLR [28].

Therefore, we conducted a scoping review to comprehensively explore existing research, clarify terminology, and identify potential pathways for the targeted design of future interventional studies, and pave the way for the application of BFR in clinical practice [29,30]. The current review aimed to address the following research questions: (1) What are the effects of BFR-RT or BFR-ET compared with non-BFR training protocols, both PRE-OP and POST-OP, in patients undergoing ACLR? (2) What are the commonly used training and BFR parameters during BFR-RT and BFR-ET protocols, both PRE-OP and POST-OP, in patients undergoing ACLR?

## 2. Materials and Methods

### 2.1. Protocol and Registration

We conducted a scoping review to explore the effects of BFR training on ACL injury and identify areas where further research is needed [31]. We aimed to understand the responses and adaptations induced by BFR in the neuromuscular, peripheral vascular, and peripheral metabolic systems. This review strictly adhered to the recommendations of the PRISMA Extension for Scoping Reviews (PRISMA-ScR) [31] and the PRISMA guidelines specific to exercise and sports science [32]. We registered the present review in Open Science Framework (1 April 2024; DOI: 10.17605/OSF.IO/76243; https://osf.io/76243/) (accessed on 1 April 2024). The preprint for this article was posted on Research Square (21 February 2025; DOI: 10.21203/rs.3.rs-6062247/v1; https://www.researchsquare.com/article/rs-6062247/latest) (accessed on 21 February 2025).

The Scoping Review design (PRISMA-ScR) was employed over a traditional Systematic Review to effectively map the breadth and characteristics of the existing evidence on BFR training after ACL injury, rather than to determine efficacy. This methodological choice was primarily driven by the substantial heterogeneity across included studies and the high diversity of outcome measures reported in the literature. As the review aimed to analyze adaptations during both the PRE-OP and POST-OP phases rather than acute responses, cross-sectional designs were excluded, while cohort studies were excluded due to the lack of intervention control and the limited comparability between BFR and non-BFR groups across both phases. Consequently, given this inherent variability and the unfeasibility of data extraction, a quantitative synthesis was deemed scientifically non-feasible.

### 2.2. Eligibility Criteria

The PICOS approach guided the selection of eligible sources, with inclusion criteria outlined in Table 1, requiring peer-reviewed original research available in full-text format, with no language restrictions applied. While scoping reviews may include observational evidence, this review focused on randomized and quasi-randomized controlled trials to ensure methodological rigor. Only studies utilizing autografts were included to ensure homogeneity and avoid confounding due to different patient profiles, healing times, and rehabilitation protocols associated with allografts.

### 2.3. Strategy of Search and Databases

Systematic literature searches were carried out in three phases. In the first phase, SWIFT-reviewer [33] was utilized for the initial exploratory search. Specific keywords such as BFR and ACL were employed to delve into, categorize, and determine the terms to utilize. In the second phase, primary searches were conducted on various databases, including Web of Science (WoS), PEDro, Scopus, PUBMED (MEDLINE), SportDiscus, and Cochrane Library databases, from inception until 24 September 2025. The search formula was adapted according to the specifics of the respective database and contained a combination of terms related to BFR and ACL injury. The search fields, filters, and results from the electronic database can be found in Table A3. In the third phase, we introduced the eligible articles from the second phase into the Citation Chaser [34]. This web platform was used to identify potential articles in forward (articles that cited the eligible articles) and backwards (references within the eligible articles) citation chasing in the literature. Additionally, a search of the gray literature was conducted via platforms such as OpenGrey, OAlster, and Google Scholar to ensure comprehensive coverage of relevant studies and reports not found in traditional academic databases. The authors were also contacted when Supplementary Material was unavailable; although some data were retrieved, incomplete responses limited data analysis.

### 2.4. Selection of Sources of Evidence and Data Extraction

The selection and screening process was conducted using Rayyan [35]. Two independent reviewers (Reviewer 1 and Reviewer 2) screened the titles and abstracts through this blinded platform [35] to assess study eligibility according to the predefined inclusion and exclusion criteria across both search phases. An experienced supervisor (Supervisor 1) was blinded to resolve conflicts in the screening process. The articles that passed the title and abstract screening were entered into the Citation Chaser platform [34] for exhaustive tracking of backwards and forward citations. Finally, the screening process was repeated for full-text review. The data extraction from the selected studies was conducted by two reviewers (Reviewer 1 and Reviewer 2), and conflicts were discussed by two supervisors (Supervisor 1 and Supervisor 2).

### 2.5. Risk of Bias, Quality of Evidence, and Quality of Exercise Reporting

The methodological quality of the RCTs and QRCTs was evaluated via the Physiotherapy Evidence Database (PEDro) [36] bias detection tool, which evaluates eleven specific criteria. The results are presented in Table A2. The Consensus on Exercise Reporting Template (CERT) [37] was used to evaluate whether the reviewed articles provided a structured framework and effectively documented and reported exercise outcomes and parameters. The results are presented in Table A3. Two evaluators independently conducted all assessments, and any disagreements were resolved through discussion and consensus with a supervisor (Supervisor 1).

### 2.6. Data Items and Synthesis of Results

To ensure terminological clarity and consistency throughout the manuscript, the following key abbreviations are used, specifically detailing the load and training conditions associated with Blood Flow Restriction (BFR): BFR-LL (BFR with Low-Load Training, 1RM); BFR-HL (BFR with High-Load Training, 1RM); BFR-RT (BFR with Resistance Training, encompassing BFR-LL and BFR-HL); BFR-ET (Blood Flow Restriction with Endurance Training) combines BFR with low-to-moderate intensity exercises—generally known as aerobic modalities such as walking, light jogging, or cycling—typically executed 40−50% VO2 max or 50% heart rate reserve; PRE-OP (Pre-Operative); and POST-OP (Post-Operative). These standardized designations are applied uniformly across the text, figures, and tables.

The study variables were categorized into six sections: body composition (including muscle volume, thickness, cross-sectional area and changes in site-specific bone mass, bone mineral density, and whole limb lean mass); neuromuscular adaptations and responses (including maximum voluntary isometric contraction, isokinetic strength, activation of vastus medialis, central activation ratio of knee extensors, and fatigue indexes); functional measurements (including all tests and values related to functionality); self-report questionnaires (including patient-reported scales and questionnaires); muscle physiology and biomarkers (including muscle biopsy analysis and blood sample analysis); and return to activity time. The results were systematically categorized and summarized into key thematic areas, and data synthesis was achieved by presenting the evidence in a narrative format complemented by detailed tables that corresponded to each outcome domain.

## 3. Results

### 3.1. Study Characteristics and Strength of Recommendations

**Figure 1:**
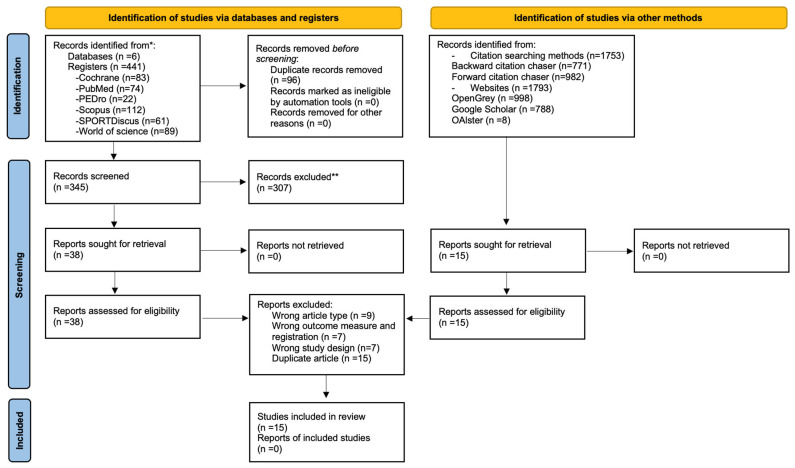
PRISMA flow chart with information on the specifics of the search process. *: It is used to indicate the sum of records identified across all databases and registers before duplicate removal. **: It is used to indicate that the specific reasons for the exclusion of these records are detailed in the body of the manuscript.

Literature searches conducted across six different databases and finalized on 24 September 2025, yielded 441 articles. After removing 96 duplicate articles and applying selection criteria to 345 articles based on title and abstract, 307 articles were excluded. A comprehensive assessment of the full texts of 38 articles was subsequently conducted, which led to the exclusion of twenty-three articles: nine were excluded because of the wrong article type, seven were omitted because of a wrong outcome measure and registration, and the remaining seven were discarded because of a wrong study design. In addition to the main search, a backwards and forward citation search identified 1753 articles, and a gray literature search yielded 1793 articles. Fifteen articles were assessed for eligibility, but all were duplicates of those found in the main search. Finally, a total of 15 studies were included in the present review. Twelve were randomized controlled trials [38,39,40,41,42,43,44,45,46], and the remaining three were quasi-randomized controlled trials [47,48,49]. The details of the charting process can be seen in Figure 1.

**Figure 2:**
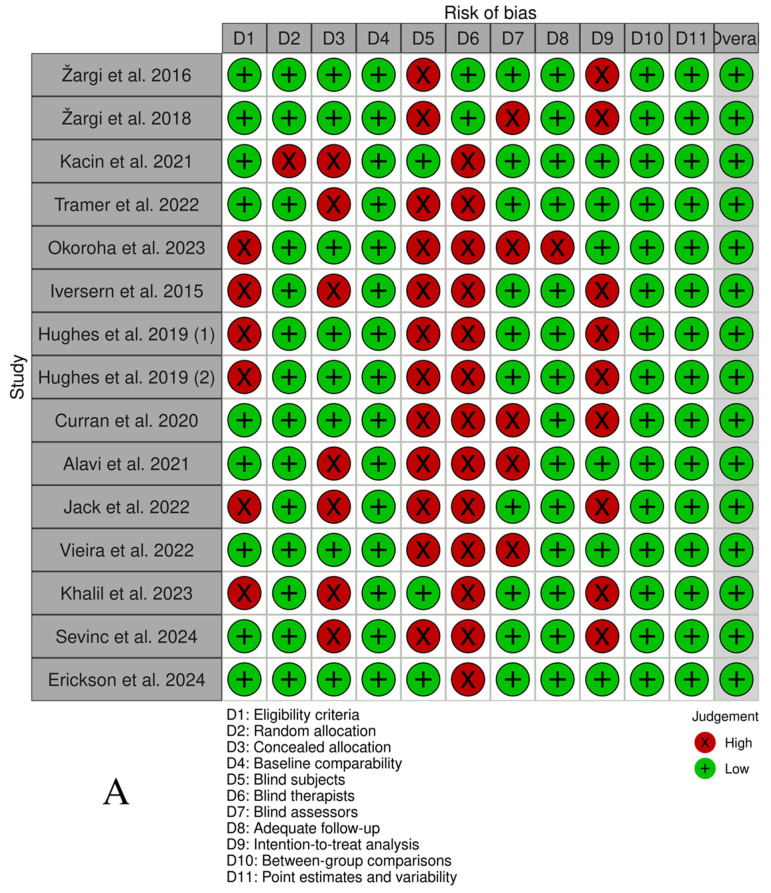

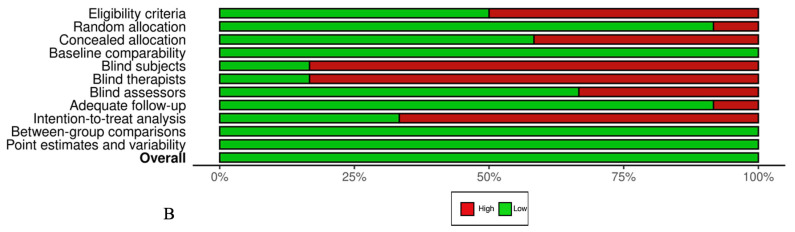
(**A**) PEDro scale by study. (**B**) PEDro scale by item (%) [38,39,40,41,42,43,44,45,46,47,48,49,50,52,53].

The overall number of articles selected demonstrated a low risk of bias according to the PEDro (36) scale, as shown in Figure 2. The median PEDro score was 6.87 (Interquartile Range[IQR]: 6–8) out of 10 for all included RCTs and QRCTs. The lowest score recorded was 6, indicating an acceptable level of quality, whereas the highest score achieved was 9, reflecting excellent quality. Most of the selected studies exhibited bias regarding blinding of all subjects (80%) and all therapists (86, 67%).

The PRE-OP articles met the specified criteria of the CERT [37] scale in 51,6% of the cases, whereas the POST-OP studies met it in 60,8% of the cases (Appendix C).

The final analysis included 417 patients who underwent ACLR (285 males and 132 females). All studies utilized BFR-RT as the experimental intervention, with one applying a cross-BFR education protocol to the uninjured leg [50] and none employing BFR-ET. Five studies used BFR-RT during the PRE-OP phase [46,47,49,51], nine were conducted POST-OP phase [40,41,42,43,44,45,50,52], and one study was performed in both the PRE- and POST-OP phases [53], which was discussed in the POST-OP results due to its longer follow-up and more extensive control during that phase. The general characteristics of included studies (time of intervention, author, study design, autograft type, intervention time, population, sample characteristics, groups, and studied variables) are shown in Table 2.

The CERT scale [37] was employed to ensure comprehensive reporting of exercise parameters (Appendix C), while the distribution of studies based on the FITT-VP parameters [54], describing exercise variables such as frequency, intensity, time, type, volume, and progression, is detailed in Appendix D. Various training protocols were employed, with training loads ranging from body weight to 70% of 1 repetition maximum, and training intensity was predominantly determined by calculating the percentage of 1 repetition maximum [40,41,42,43,47,48,49]. Intermittent [43,44,46] and continuous [38,40,41,42,45,50] rest periods were utilized, with various tools employed for calculating limb occlusion pressure (LOP). No adverse events were reported during interventions [38,40,41,42,45,46]. Specific training parameters and cuff details for individual studies are outlined in Table 3.

### 3.2. Outcome Measures

A summary of evidence from the selected studies on BFR protocols across the continuum from prehabilitation to post-ACLR rehabilitation is provided in Table A4. Tier List of Clinical Outcomes: Efficacy of BFR Training in ACLR. This table represents reported outcomes and should not be interpreted as a clinical guideline.

#### 3.2.1. Body Composition

The PRE-OP interventions demonstrated improvements [47] or comparable [38,46] results in quadriceps cross-sectional area (CSA) between BFR-RT groups and comparison groups. In POST-OP studies, BFR-LL demonstrated comparable CSA effects to those of HL-RT at 70% 1RM [41,45,53], yet combining BFR with HL (BFR-HL) yielded no additional benefits [43]. However, combining BFR-RT with home-based interventions with body-weighted isometric protocols appeared ineffective [44]. Jack et al. [42] observed a protective effect of BFR-LL against post-ACLR bone loss and lean mass compared with LL-RT.

#### 3.2.2. Neuromuscular Adaptations and Responses

PRE-OP interventions significantly improved knee extensor peak torque [38,47] or yielded similar results [48,49] compared with LL-BFR or Sham-BFR groups. A PRE-OP research team made significant progress in their studies [47,48,49], consistently achieving favorable outcomes within the BFR-LL group and effectively preventing the deterioration of maximal muscle strength and knee extensor endurance.

Hughes et al. (2019b) [41] POST-OP study compared BFR-LL at 30% and HL-RT at 70%. At 8 weeks, both groups showed similar increases in scaled 10RM strength and similar decreases in knee extension peak torque. However, the HL-RT group presented significantly greater decreases in knee flexion peak torque at all speeds than the BFR-LL group. This was accompanied by a strong effect size, suggesting a more detrimental effect for HL-RT. Vieira et al. [45] utilized similar groups and reported that, compared with HL-RT, BFR-LL resulted in significantly greater improvements in muscle strength within a shorter rehabilitation period, whereas Curran et al. [43] demonstrated that HL-RT alone resulted in significant improvements in quadriceps muscle strength, without any additional benefit from incorporating BFR into HL-RT. Additionally, Erickson et al. [53] reported that BFR-LL was equally effective as Sham-BFR combined with HL-RT in their PRE- and POST-OP studies. Ultimately, Sevinc et al. [50] reported a significant main effect of time on quadriceps strength in both the involved (*p* < 0.001) and uninjured limbs, with no additional gains from adding BFR to cross-education in post-ACLR patients.

#### 3.2.3. Self-Report Questionnaires

Self-report questionnaires were used in seven studies to evaluate knee pain, muscle pain, rate of perceived exertion, knee function, knee symptomatology, and self-perceived depression [38,39,40,41,43,45,46]. Home-based PRE-OP interventions yielded similar [46] or improved [38] outcomes between BFR-LL and LL-RT. However, in POST-OP interventions, BFR-RT consistently improved knee joint pain and self-reported outcomes in studies comparing BFR-LL vs. HL-RT [40,41,45], except for muscle pain immediately following each set of exercise [40], where the BFR-LL group had worse values than did the HL-RT group. Additionally, the study of Khalil et al. [39]. compared BFR-LL vs. LL-RT, and another study comparing BFR-HL (70% 1 RM) vs. HL-RT [43] did not yield significant improvements.

#### 3.2.4. Functional Measurements

Three PRE-OP studies investigated functional measurements but did not find significant improvements in knee ROM [38,46] or Y-balance tests [49]. However, in contrast, POST-OP interventions demonstrated significant results. Hughes et al. (2019b) [41] reported significant improvements in Y-balance on the injured leg across all directions (anterior, posteromedial, and posterolateral), with the BFR-LL group exhibiting a strong effect size compared with the HL-RT group. Additionally, in the study by Jack et al. [42] significant improvement in Y balance was observed only in the BFR-LL group between 8 and 12 weeks compared with the LL-RT group.

Hughes et al. (2019b) [41] demonstrated significant improvements in mid-patellar knee joint circumference and knee range of motion (ROM), with no observed changes in knee laxity in the BFR-LL group.

#### 3.2.5. Muscle Physiology and Biomarkers

In the PRE-OP study by Žargi et al. [49], significant improvements in muscle blood flow were observed at week 4 POST-OP in the BFR-LL group (↑52%) compared with the Sham-BFR group (↓37%), with a notable interaction detected between time and group factors in muscle blood flow. Additionally, Kacin et al. [47] conducted a biopsy analysis, emphasizing the role of hypoxia-inducible factor 1-alpha (HIF-1α) in cellular adaptation to hypoxia. These authors reported higher mRNA levels of VEGF-A, which is crucial for angiogenesis regulation, in the BFR-LL group than in the Sham-BFR group.

Regarding the POST-OP phase, one study [52] examined key biomarkers related to muscle atrophy following ACLR through blood samples. The results revealed significant reductions in serum Atrogin-1 (↓−12.53%) and serum MuRF1 (↓−15.47%) levels from week 0 to week 12 in the BFR-RT group compared with those in the RT group, with a significant group × time interaction for Atrogin-1 and a significant time effect for both biomarkers (*p* < 0.05).

#### 3.2.6. Return to Activity Time

Jack et al. [42] demonstrated a significant reduction in the time to return to sports by 1.4 months, accompanied by a strong effect size, in a 12-week rehabilitation protocol when BFR-LL was compared with LL-RT. Curran et al. [43] found no significant differences between BFR-HL and HL-RT.

### 3.3. Exercise Parameters and Reporting Standards

The CERT scale [37] was used to evaluate the practical implementation and completeness of exercise reporting across the included studies. In our analysis, adherence to the CERT criteria was 51.6% for PRE-OP interventions and 60.8% for POST-OP interventions. Notably, compared with PRE-OP studies, POST-OP studies achieved equal or better results for 13 of the 19 evaluable criteria. Both PRE-OP and POST-OP studies presented low scores in detailing how adherence to exercise is measured and reported (PRE-OP: 0% vs. POST-OP: 42.9%). In contrast, both types of articles provided a detailed description of each exercise to enable replication (PRE-OP: 100% vs. POST-OP: 88.9%). PRE-OP studies accounted for the detailed description of any home program component in 100% of cases, whereas POST-OP studies did so in 55.6% of cases. Finally, 77.8% of POST-OP studies explained how exercises are tailored to the individual, whereas none of the PRE-OP studies reported this. One article that examined both the PRE- and POST-OP phases achieved favorable results in 13 of the 19 criteria. To prevent influencing the outcomes, the studies were analyzed separately for each phase.

**Figure A1:**
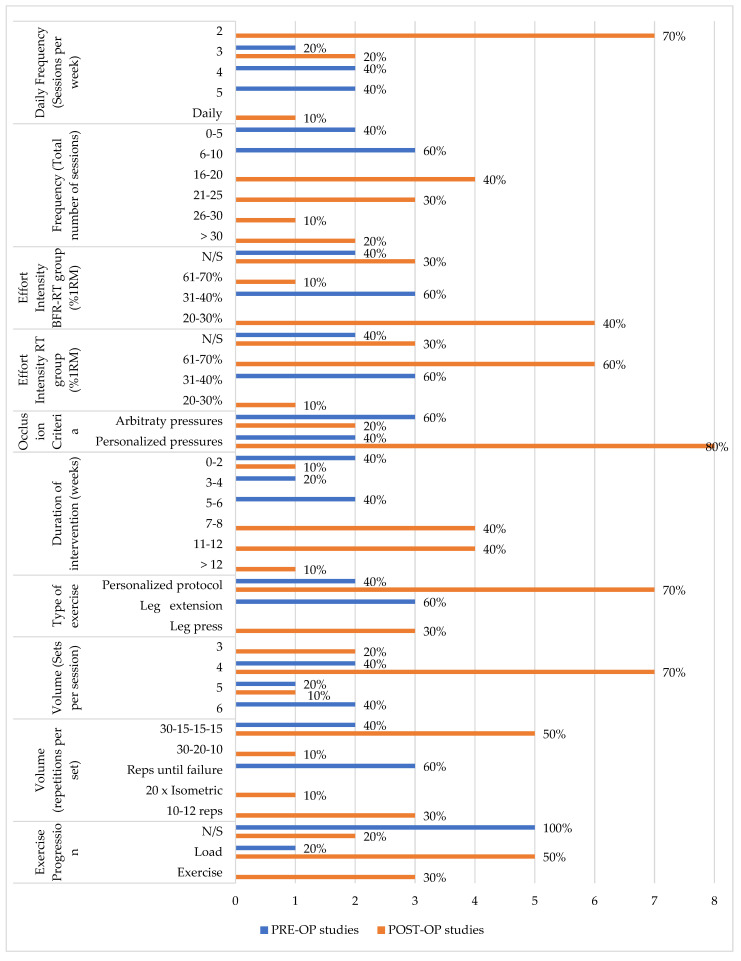
Distribution of the studies according to the FITT-VP parameters. Data given as the total number of protocols represented. The percentage shown reflects the absolute percentage of studies using each parameter within the respective PRE-OP or POST-OP category. The study by Erickson et al. [53], covering both PRE- and POST-OP phases, was included in POST-OP for clarity and space considerations. Categories without values were omitted for conciseness. Abbreviations: POST-OP, postoperative; PRE-OP, preoperative; RM, repetition maximum; BFR-RT, blood flow restriction resistance training; RT, resistance training; mmHg, millimeters of mercury; N/S, not specified.

The reporting of exercise parameters on the FITT-VP [54] framework revealed variability between studies in both phases (Figure A1). PRE-OP studies included short-duration interventions, averaging 1.91 weeks with 5–10 sessions. In contrast, POST-OP studies utilized longer protocols, with all studies having 16 or more sessions and an average intervention duration of 9 weeks. Additionally, the studies that examined both PRE- and POST-OP ACLR phases had a duration of 24 weeks, the longest among all those analyzed. A total of 9 studies from the review utilized personalized exercise protocols. (PRE-OP: 2/5 vs. POST-OP: 6/9 vs. PRE- and POST-OP: 1/1), whereas the remaining studies focused predominantly on specific exercises (PRE-OP: 3/5 for leg extension vs. POST-OP: 3/9 for leg press). Regarding repetitions, adherence to the “30-15-15-15” protocol was noted in 40% of PRE-OP studies compared with 55.5% of POST-OP studies. Additionally, the remaining PRE-OP studies aimed for repetitions to failure, whereas the POST-OP studies that did not follow the “30-15-15-15” protocol adhered to different repetition patterns, similar to the study that examined both phases and followed its rehabilitation plan. Among the studies reviewed, 66.7% determined the optimal exercise load by one repetition maximum. Training loads for the BFR-RT group ranged from body weight to 30% of 1RM, except for two studies that employed 70% of 1RM. Progression of training stimulus was reported in 2/5 of PRE-OP interventions compared with 8/9 of POST-OP studies and PRE- and POST-OP studies 1/1.

## 4. Discussion

In this scoping review examining the application of BFR-RT before and after ACLR, no study reported that BFR-RT was less effective than the comparison group in any outcome measure, except for one specific study where knee pain during training sessions was greater in the BFR-LL group than in the HL-RT group. Specifically, 2/5 of PRE-OP studies reported superior outcomes in BFR-RT interventions compared with groups trained with the same or different loads. Among the POST-OP interventions, 5/9 studies reported superior results for BFR-RT, with the remaining studies reporting comparable outcomes between the BFR-RT and comparison groups. None of the studies included BFR-ET, highlighting the need for future research on its effectiveness in ACLR rehabilitation. It is plausible that studies prioritize BFR-RT due to the existing body of evidence and clinical consensus supporting its efficacy in mitigating atrophy and maximizing strength recovery in early rehabilitation phases.

Based on the evidence reviewed in the PRE- and POST-OP phases of ACLR, BFR-RT shows promise as a useful interim step [55]. The results of our review regarding body composition, neuromuscular adaptations, muscle physiology and biomarkers, and self-report questionnaires are consistent with those reported in other systematic reviews investigating the effects of BFR on ACLR [56,57,58]. Furthermore, we emphasized rigorous methodological evaluation via the PEDro scale [36]. Additionally, we employed the CERT scale [37] to assess the quality of reporting exercise parameters. These tools are crucial for extracting reliable information related to exercise parameters and methodology, ensuring that study results are applicable in practical settings. On the other hand, previous reviews have incorporated non-randomized trials and studies with a high risk of bias [26,27,57,58], which can affect the reliability of the findings. To avoid such biases, we included only RCTs and QRCTs in our review. Our review stands out from previous reviews because of its emphasis on precise article selection criteria, the inclusion of high-quality trials, and the meticulous extraction of information concerning exercise parameters and BFR methodology. This is the first systematic review that specifically evaluated the use of BFR combined with exercise during PRE-OP and POST-OP in ACLR patients.

PRE-OP interventions were specifically designed to explore the effects of BFR-RT on muscle preservation, strength maintenance, and overall PRE-OP outcomes. Factors such as immobilization, restricted weight bearing, intraoperative tourniquet and nerve block administration during ACLR surgery may contribute to quadriceps weakness [11]. This weakness can impact functionality [59,60], quality of life [11,61], and joint health over time [62]. In other reviews, exercise-based prehabilitation has been shown to be favorable for ACLR [63,64], whereas adding BFR to prehabilitation appears promising [26]. However, Žargi et al. [49] The initial study implemented BFR-LL with knee extension exercises for 10 days PRE-OP but reported no significant POST-OP effects compared with LL-RT in terms of maximum voluntary isometric contraction and CSA of knee extensors. Other studies highlighted that quadriceps endurance, rather than maximal strength and CSA, emerged as the most significant predictor of quadriceps atrophy following ACLR [19,51]. Following the PRE-OP protocol established in the study by Zargi et al. [48], two new studies [47,49] selected different outcomes while maintaining the original intervention protocol. A statistically significant group interaction was detected at week 4 POST-OP, with near-infrared spectroscopy and muscle surface electromyography activation [48]. The second PRE-OP study highlighted statistically significant improvements in the muscle fatigue index [47]. At 3 weeks POST-OP, the BFR-LL group presented a 60% decrease in peak torque at 60°/s, whereas the LL-RT group presented a 21% decrease. The previous results confirm findings in other systematic reviews [65], were improvements related to muscle endurance and blood flow muscle parameters may prevent ischemia—reperfusion damage and protect against muscle protein oxidation [65,66], and consequently, these improvements may serve as a safeguard against the threat of POST-OP quadriceps atrophy [26]. Moreover, the PRE-OP study of Kacin et al. [47] conducted muscle biopsies in ACLR patients and underlines a significant impact of BFR-RT on vascular endothelial growth factor-A (VEGF-A) and its mRNA levels, which are crucial for angiogenesis [65]. These results align with trends from other reviews based on the general population [65,66]. Identifying effective strategies to increase muscle endurance could optimize both PRE- and POST-OP results.

POST-OP interventions using BFR-RT are typically implemented in the early phases after ACLR. These interventions aim to investigate the impact of BFR-RT on muscle recovery, strength restoration, and overall POST-OP rehabilitation in individuals who have undergone ACLR. Specifically, improvements in muscle volume, a key aspect of body composition, were observed and align with findings from other systematic reviews, highlighting the potential of BFR-RT in enhancing muscle recovery and rehabilitation outcomes [11,27]. BFR-LL has been demonstrated to be superior to LL-RT [42] and comparable to HL-RT [41,45] in enhancing muscle volume. Moreover, BFR-HL versus HL-RT yielded similar results [43]; notably, BFR-HL may deviate from the fundamental physiological principles of BFR-RT, as it involves HL-RT rather than the LL approach typically used in BFR-RT. Additionally, a POST-OP study [52] analyzed two key biomarkers of muscle atrophy, Atrogin-1 and MuRF1, which are typically elevated in response to disuse. The study underlines significant reductions in serum Atrogin-1 (↓12.53%) and MuRF1 (↓15.47%) levels in the BFR-RT group compared with those in the RT group, suggesting that BFR may help mitigate muscle protein degradation and prevent atrophy.

Parameters related to bone mineral density were examined in the study by Jack et al. [42] These significant findings align with the meta-analysis by Wang et al. [67], highlighting that BFR-LL training results in greater improvements in bone health than does LL-RT [68]. Better bone mineralization may enhance graft integration in ACLR procedures and help prevent conditions such as osteopenia and osteoporosis in specific patients, making this a relevant line of investigation for future research.

A previous meta-analysis [69] focusing on non-injured populations demonstrated that, compared with BFR-LL, HL-RT leads to greater muscle strength gains. However, our review underlines that, compared with HL-RT, BFR-LL or BFR-HL interventions produce similar or superior muscle strength [42,44,46] in ACLR patients [41,43,45]. Additionally, a study combining BFR with isokinetic training and cross-education on the uninjured leg found no additional benefits from adding BFR, as both groups achieved similar results [50]. Comparing BFR-RT to approaches such as BFR-HL or BFR with isokinetic training may have deviated from the fundamental physiological principles of BFR-RT. Moreover, some of these studies concluded that BFR-LL was ineffective simply because it produced similar outcomes to HL-RT, overlooking the fact that achieving comparable results with significantly lower loads is, in itself, a meaningful finding. This misinterpretation may stem from methodological choices, particularly the use of HL-RT ‘sham groups’ and the selection of comparators, which could have influenced the perceived effectiveness of BFR. During the early to middle stages of POST-OP ACLR, BFR-RT has emerged as an effective strategy, particularly when HL-RT may cause pain or when limited mobility or high-load intolerance is present.

Knee pain is a key outcome of ACLR and BFR-RT research. BFR-LL has been shown to facilitate a more rapid reduction in knee pain than does HL-RT, which is correlated with improved functionality and quality of life [40,41,45]. The physiological mechanisms behind BFR-RT induced pain reduction are not fully understood, but several theories have been proposed. These include activation of the opioid and endocannabinoid systems, increased activation of the descending inhibitory pathway, early preferential recruitment of high-threshold motor units (Type II), and conditioned pain modulation, where pressure and discomfort during BFR-RT may act as conditioning stimuli [68,70,71]. However, further research is needed to fully understand and validate these mechanisms to establish viable pain management protocols.

In our review, home-based BFR rehabilitation unsupervised exercise protocols [38,44,46] generally demonstrated less significant improvements than supervised protocols did [39,40,41,42,43,45,47,48,49]. This contrasts with findings from other reviews that combine exercise without BFR, suggesting that supervision and location do not directly determine final outcomes in ACL rehabilitation [72,73,74]. Self-application of BFR cuffs requires precise pressure settings and effective discomfort management, which may impact adherence and treatment effectiveness. Identifying key factors that enhance home-based interventions could be pivotal in optimizing their efficacy and reducing the socioeconomic costs associated with ACLR rehabilitation.

When evaluating the practical implementation and training parameters in experimental interventions via CERT scale [37], we identified a significant disparity. This inconsistency arises from the lack of a standardized framework for describing exercise parameters (FITT-VP) [54] and BFR-RT methods. PRE-OP interventions for ACLR adhered to CERT criteria in 51.6% of the parameters, whereas POST-OP interventions followed them in 63.9%. This difference likely reflects a more developed research line in the POST-OP phase. However, PRE-OP interventions are being increasingly investigated [75], with a focus on short-term adaptations to mitigate iatrogenic atrophy from the injury and surgery, ultimately aiming to “prepare the leg for the storm” [64]. This contrasts with the longer interventions reported in the POST-OP phases. The shorter duration of PRE-OP interventions may be associated with the limited time before surgery, which is often scheduled within weeks [76]. Although consensus on specific prehabilitation protocols for BFR-RE and ACLR remains limited, emerging research suggests that extended prehabilitation could lead to improved long-term outcomes in certain cases [77]. Notably, all PRE-OP studies incorporated open kinetic chain (OKC) exercises, whereas POST-OP studies predominantly utilized closed kinetic chain (CKC) exercises. This could be due to some articles suggesting that OKC exercises, such as single-leg extensions, may place significant strain on the ACL graft because of the lack of co-contraction between the quadriceps and hamstrings, potentially resulting in excessive anterior shear forces on the knee joint [78,79]. Meta-analyses have shown no clear superiority between open and closed kinetic chain exercises after ACL reconstruction regarding knee laxity and overall outcomes [79,80]. In both open and closed kinetic chain exercises, load progression is crucial for effective rehabilitation. The reviewed articles suggest that BFR-LL may be beneficial in early ACLR phases, providing a safe way to increase loading. Beyond exercise modality, a critical analytical disparity exists in BFR prescription parameters. PRE-OP protocols primarily relied on fixed, arbitrary cuff pressures or used LOP with fewer or no re-measurements during the intervention [47,48,49], representing a significant methodological limitation. Conversely, POST-OP protocols show a strong methodological shift toward individualized pressure settings, reflecting the consensus that BFR pressure should be individualized relative to LOP for safety and efficacy. In addition, most POST-OP trials specified the loads applied and described their progression, while PRE-OP interventions, often of shorter duration, lacked such detail or load progression. Clinically, while PRE-OP BFR-LL mitigates atrophy, its future implementation requires LOP-based pressure prescription to meet precision standards. PRE-OP studies are generally older, which may have limited the frequency and precision of LOP measurements compared with more recent POST-OP studies, where automatic devices were available. This disparity ultimately highlights the need for standardized protocols and clear, evidence-based guidelines to inform future ACLR rehabilitation frameworks.

Given the pivotal role of exercise parameters in optimizing rehabilitation outcomes, the notion that exercise functions as a medicine highlights the necessity of understanding its appropriate dosage and administration [81]. The lack of replicability in exercise parameters observed in our review is not unique; other reviews involving diverse pathologies [82] or PRE-OP ACLR patients [27,83] have shown similar inconsistencies. Reporting these parameters is crucial for enhancing the replicability and transferability of findings to clinical practice. Consistent with the overall lack of reporting, none of the studies in our review that reached muscular failure reported on effort intensity relative to muscle failure proximity. This omission has physiological implications, as effort intensity can significantly influence physiological responses and adaptations. The studies included in our review yielded results such as systematic reviews examining training for muscular failure, which increases metabolic response, muscle damage, and perceived exertion while decreasing biomechanical properties [84,85]. Nonetheless, this approach provides gains in strength and muscle size comparable to non-failure training. Notably, applying BFR-LL to volitional failure yields similar results to LL-RT, but BFR-LL leads to earlier failure than does LL-RT [45,86], reducing session volume [87] and duration. This makes BFR-LL a valuable option when reaching failure, which is an objective during rehabilitation stages.

With respect to fatigue, intermittent BFR-RT has demonstrated strength gains comparable to continuous application but with reduced fatigue [88,89]. However, only one study utilized intermittent BFR-RT [43]. Our scoping review, in contrast with previous reviews [42,90], highlights a shift from using arbitrary pressures to more reliable methods for determining LOP, autoregulated devices, with automatic LOP measurement capability [91,92]. Ensuring optimal LOP is crucial for the efficacy and safety of BFR combined with exercise [56,91,92]. None of the studies included in our review reported adverse events, supporting Bond’s assertion of a low risk of thromboembolism associated with BFR [92]. Notably, our study population typically excludes individuals at high risk for thromboembolism. To increase safety, clinicians should thoroughly screen for signs of venous thromboembolism, assess individual risks, and implement appropriate protocols when integrating BFR.

### 4.1. Limitations

Our scoping review identified several limitations in the current scientific literature on BFR combined with exercise and ACLR, as summarized in Table 4. 

A notable gap exists in studies applying BFR+ET for ACLR. The considerable heterogeneity in study designs complicates direct comparisons, whereas the limited number of studies affects overall comprehensiveness. Variations in BFR combined with exercise interventions, outcome measures, and exercise parameters significantly influence outcomes. Additionally, diverse measures and non-standardized follow-up further complicate synthesis and long-term effect assessment. The complexity of blinding of therapists and subjects may introduce bias, and the lack of sham situations and intention-to-treat analyses further undermines the robustness of the findings. Research should focus on developing and validating effective sham/placebo BFR conditions and rigorously documenting and justifying blinding strategies to enhance internal validity. Poor reporting of exercise-related parameters also necessitates caution when interpreting results. Owing to the diversity of outcomes and parameters, conducting a meta-analysis was not feasible, and in some cases, data extraction from articles [39,40,41,43,46] was not possible. A limitation of our review is the potential exclusion of relevant studies due to the omission of certain databases in the search process. Therefore, further studies are needed to explore long-term effects, adaptations, recovery, and reinjury rates of both PRE- and POST-OP interventions. To draw more definitive conclusions, further research with improved methodologies and patient follow-up is essential. Despite these challenges, the current literature underscores the growing importance of BFR-RT during PRE- and POST-OP phases for ACLR.

### 4.2. Future Directions

Future research on BFR in ACLR rehabilitation should prioritize the establishment of standardized BFR-RT protocols with precise reporting of FITT-VP parameters, cuff characteristics, and adherence, in order to identify the most effective and clinically applicable approaches. Large randomized controlled trials with extended follow-up are needed to evaluate long-term adaptations, reinjury risk, and the durability of functional outcomes. The lack of studies on BFR-ET represents an important gap, as endurance-oriented applications may elicit adaptations in muscular endurance and other physiological responses relevant to ACLR recovery. Finally, further research is required to clarify the physiological responses and adaptations to BFR to strengthen the evidence base and guide its clinical integration.

## 5. Conclusions

The evidence examined demonstrates that the use of BFR-RT for ACLR holds significant promise in both PRE- and POST-OP phases. In the PRE-OP phase, BFR-RT application significantly increased muscle strength and endurance and improved parameters linked to angiogenesis and transcriptional responses in comparison with LL-RT. Postoperatively, notable improvements in the BFR-RT group were observed in self-report questionnaires, knee pain, muscle volume, and muscle strength. When BFR-LL was compared with HL-RT, BFR-LL showed similar or greater improvements in functional measurements, muscle hypertrophy, and strength, although it also reported greater muscle pain during training. Home-based BFR-LL interventions were feasible and well tolerated by patients.

These findings support the integration of BFR-RT into clinical practice in both the pre- and early post-ACLR rehabilitation phases. In the preoperative phase, BFR-RT can contribute to preserving muscle function and preparing patients for surgery, while in the postoperative phase, it can be applied when weight-bearing activities are limited to increase muscle strength and endurance and minimize the risk of disuse atrophy. Clinicians should adopt a patient-centered perspective, tailoring BFR interventions to individual needs and ensuring both safety and comfort to enhance optimal rehabilitation.

## Figures and Tables

**Table 1:** Inclusion and exclusion criteria based on the PICOS strategy.

	Inclusion Criteria	Exclusion Criteria
Population	Healthy human patients affected by a primary ACL injury with or without concomitant meniscal procedures and surgically intervened exclusively with autograft (patellar, quadriceps, or hamstring tendon) were included.	Previous ACL injury, additional knee impairments, multiple reconstructive procedures, or those treated with allografts.
Intervention	Interventions that combined BFR and resistance exercises or endurance exercises applied during PRE- and/or POST-OP phases. Endurance exercises, including common aerobic modalities such as cycling or treadmill walking, were specifically included when combined with BFR (BFR-ET). The duration of the intervention should be at least one week for both PRE- and POST-OP.	Analyses were not conducted independently for interventions combining BFR with techniques such as electrostimulation or other modalities, or the duration of the interventions was lower than required in the inclusion criteria.
Comparator	Comparison group performing resistance exercises or endurance exercises with or without BFR or a placebo (i.e., Sham-BFR).	There is no control/comparison group. Articles that do not contain a comparative analysis with control/comparison groups without BFR during exercise interventions, or a placebo (i.e., simulated BFR).
Outcomes	Validated assessment analyzing muscular structural changes, muscle strength, neuromuscular adaptations and responses, functional changes, self-report questionnaires, pain, peripheral vascular and local metabolic parameters.	No reported validated tests providing quantitative values.
Study design	Randomized controlled trials or quasi-randomized controlled trials with pre-post measurements of one or more outcomes, comparing BFR to a control or comparison group.	Opinion articles, editorials, systematic reviews, case–control studies, case series studies, conference abstracts, in-progress articles, cohort articles, cross-sectional articles, and studies with results obtained without an initial evaluation.

ACL, anterior cruciate ligament; BFR, blood flow restriction; PRE-OP, preoperative; POST-OP, postoperative; QRCT, quasi-randomized controlled trial; RCT, randomized controlled trial.

**Table 2:** General characteristics of selected studies.

Study Features ^a^	Sample Characteristics	Intervention		Comparator Group	Study Variables					
		Autograft Type	Intervention Time		NMAR	BC	FM	MBF	SRQ	RTA
**Žargi et al.** [49] **Slovenia 2016** **PRE-OP QRCT**	N = 20(M16/W4); Age (y): IG 33 ± 7, CG 34 ± 10; BMI (kg/m^2^): IG 23.7 ± 3.1, CG 23.5 ± 3.9	HS	10 days prior to surgery.	Comparison Sham-BFR group.	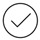	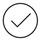	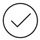			
**Žargi et al.** [48] **Slovenia 2018** **PRE-OP QRCT**	N = 20(M16/W4); Age (y): IG 34 ± 6, CG 35 ± 5; BMI (kg/m^2^): IG 24.3 ± 3.9, CG 23.9 ± 2.9	HS	8 days prior to surgery.	Comparison Sham-BFR group.	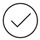			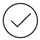		
**Kacin et al.** [47] **Slovenia 2021** **PRE-OP QRCT**	N = 18 (M9/W9); Age (y): IG 38 ± 6, CG 38 ± 8, CG(I) 36 ± 9; BMI (kg/m^2^): IG 26.0 ± 4.9, CG 25.8 ± 5.4, CG(I) 24.8 ± 3.2	HS	3 weeks.	Comparison Sham-BFR group and CG for muscle biopsy analysis.	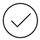	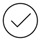				
**Tramer et al.** [46] **United States 2022** **PRE-OP RCT**	N = 45 (M25/W20); Age (y): IG 26.5 ± 12.0, CG 27.0 ± 11.0; BMI (kg/m^2^): IG 25.3 ± 3.2, CG 26.8 ± 4.9.	BTB(34), HS (13), QT (3)	14 days prior to surgery.	Comparison group without BFR.	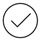	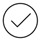	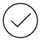		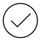	
**Okoroha et al.** [38] **United States 2023** **PRE-OP RCT**	N = 46 (M28/W18); Age (y): IG 25.4 ± 10.6, CG 27.5 ± 12.0; BMI (kg/m^2^): IG 24.9 ± 3.1, CG 26.9 ± 5.3.	BTB (13), HS (7), QT (2)	14 days prior to surgery.	Comparison group without BFR.	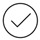	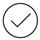			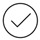	
**Iversern et al.** [44] **POST-OP** **China 2015** **POST-OP RCT**	N = 24 (M14/W10); Age (y): IG 24.9 ± 7.4, CG 29.8 ± 9.3; BMI (kg/m^2^): N/S.	HS	14 days.	Comparison group without BFR.		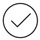				
**Hughes et al. (2019a)** [40] **England 2019** **POST-OP RCT**	N = 24 (M17/W7); Age (y): IG 29 ± 7, CG 29 ± 7; BMI (kg/m^2^): IG 25.4 ± 3.9, CG 26.4 ± 4.4.	HS	8 weeks.	Comparison group HL-RT without BFR.					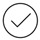	
**Hughes et al. (2019b)** [41] **England 2019** **POST-OP RCT**	N = 24 (M17/W7); Age (y): IG 29.0 ± 7.0, CG 29.0 ± 7.0; BMI (kg/m^2^): IG 25.4 ± 3.9, CG 26.4 ± 4.4.	HS	8 weeks.	Comparison group HL-RT without BFR.	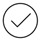	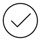	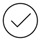		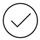	
**Curran et al.** [43] **United States** **2020** **POST-OP RCT**	N = 34 (M15/W19); Age (y): CGcon 16.1 ± 2.6, IGcon 18.8 ± 3.9, CGecc 15.3 ± 0.9, IGecc 16.0 ± 1.7; BMI (kg/m^2^): N/S.	BTB(25), HS (6), QT (3)	8 weeks.	Comparison group concentric and eccentric HL-RT without BFR.	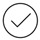	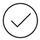			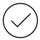	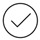
**Alavi et al.** [52] **Iran 2021** **POST-OP RCT**	N = 20 (M20/W0; Athletes); Age (y): IG 24.1 ± 4.9, CG 25.2 ± 2.6; BMI (kg/m^2^): IG 22.2 ± 6.4, CG 22.3 ± 8.6.	N/S	12 weeks.	Comparison group without BFR.		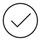				
**Jack et al.** [42] **United States 2022** **POST-OP RCT**	N = 32 (M17/W15); Age (y): IG 28.1 ± 7.4, CG 24.1 ± 7.2; BMI (kg/m^2^): IG 25.2 ± 2.8, CG 26.9 ± 5.3.	BTB	12 weeks.	Comparison group without BFR.		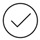	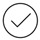			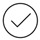
**Vieira et al.** [45] **Brasil 2022** **POST-OP RCT**	N = 24 (M17/W7); Age (y): IG 41.1 ± 9.8, CG 39.6 ± 10.8; BMI (kg/m^2^): IG 24.2 ± 3.0, CG 23.6 ± 2.4.	HS	12 weeks.	Comparison group without BFR.	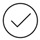				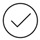	
**Khalil et al.** [39] **United States 2023** **POST-OP RCT**	N = 36 (M31/W5); Age (y): IG 23.8 ± 3.9, CG 25.2 ± 4.8; BMI (kg/m^2^): IG 26.2 ± 3.9, CG 25.4 ± 2.1.	HS	11 weeks	Comparison group without BFR.					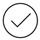	
**Sevinc et al.** [50] **Turkey 2024** **POST-OP RCT**	N = 24 (M24/W0); Age (y): IG 24.7 ± 7.0, CG 25.3 ± 5.7; BMI (kg/m^2^): IG 24.1 ± 2.4, CG 23.9 ± 2.5.	HS	8 weeks	Comparison group without BFR.	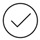	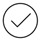				
**Erickson et al.** [53] **United States** **2024** **PRE- AND** **POST-OP RCT**	N = 48 (M28/W20, Athletes); Age (y): IG 21.1 ± 6.3, CG 21.5 ± 5.3; BMI (kg/m^2^): IG 24.9 ± 3.9, CG 25.7 ± 4.5.	BTB(45), HS (3)	24 weeks	Comparison Sham-BFR group.	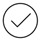	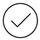	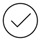			

Note: ✓ indicates that the outcome was assessed in the study. ACLR, anterior cruciate ligament reconstruction; BC, body composition; BFR, blood flow restriction; BMI, body mass index; BTB, bone-patella tendon-bone autograft; IG, intervention group; CG, comparison group; FM, functional measurements; HL-RT, heavy load resistance exercise; HS, hamstrings tendon; M, man; MPB, muscle physiology and biomarkers; N, sample number; NMAR, neuromuscular adaptations and responses; N/S, not specified; POST-OP, postoperative; PRE-OP, preoperative; QRCT, quasi-randomized controlled trial; QT, quadriceps tendon; RCT, Randomized controlled trial; RTA, return to activity; SRQ, self-report questionnaires; W, woman; y.o, years old. ^a^ Author, country, year, time of intervention, design.

**Table 3:** Exercise training doses and BFR parameters used in the selected studies.

Study Features ^a^	Exercise Training Prescription		Cuff Parameters	Outcomes	
	Intervention Schedule	FITT-VP	Device and Pressure	Measures	Within and Between Changes and Summary
**Žargi et al.** [49] **Slovenia** **2016** **QRCT**	PRE-OP Start: ten days PRE-OP. End: 48 h PRE-OP.	Freq: 3–4/wk (10 d); 5 sessions. Int: 40% 1RM, to volitional failure. Type: Single-leg knee ext. (OKC). Vol: 6 × failure. Rest: 45 s (sets 1,3,5, cuff on); 90 s(sets 2,4, cuff off). Prog: N/S.	Device: Pneumatic occlusion cuff (14 cm, contoured). Pressure: BFR-LL 150 mmHg; Sham-BFR 20 mmHg (not adjusted in real time). LOP: Arbitrary.	Isometric knee extension (KE): Maximal voluntary isometric contraction (MVIC, N). MRI: Cross-sectional area (CSA) of KE (cm^3^). Balance test: Anterior reach of star excursion; neuromuscular control (cm).	Summary: No sig. changes in short-term BFR-LL on POST-OP.
**Žargi et al.** [48] **Slovenia** **2018** **QRCT**	PRE-OP Start: eight days PRE-OP. End: 48 h PRE-OP.	Freq: 3–4/week (8 days); 5 sessions. Int: 40% 1RM, to volitional failure. Type: Single-leg knee extension (OKC). Vol: 6 × failure. Rest: 45 s (sets 1,3,5, cuff on); 90 s (sets 2,4, cuff off). Prog: N/S.	Device: Pneumatic occlusion cuff (14 cm, contoured). Pressure: BFR-LL 150 mmHg; Sham-BFR 20 mmHg (not adjusted in real time). LOP: Arbitrary.	Isometric KE: MVIC (N). EMG: Vastus medialis activation (mV). NIRS: Vastus lateralis muscle blood flow (mL·min^−1^·100 mL^−1^).	Time to contraction (wk 4 POST-OP): # IG ↑6%; # CG ↓51%. Time to contraction (wk 12 POST-OP): # IG ↑37%; # CG ↓3%. Sig. group interaction (*p* < 0.014, d = N/S). RMS-EMG amplitude (wk 4 POST-OP): # IG ↑43%; # CG ↓17%. RMS-EMG amplitude (wk 12 POST-OP): # IG ↓7%; # CG ↓6%. Sig. group interaction (*p* < 0.001, d = N/S). Muscle blood flow (wk 4 POST-OP): *,# IG ↑52%; *,# CG ↓37%. Muscle blood flow (wk 12 POST-OP): *,# IG ↑18%; *,# CG ↓11%. Sig. group interaction (*p* < 0.001, d = N/S). Summary: Short-term BFR-LL ↑ quadriceps endurance, fiber recruitment, and perfusion after ACLR.
**Kacin et al.** [47] **Slovenia** **2021** **QRCT**	PRE-OP Start: three weeks PRE-OP. End: N/S.	Freq: 3/week (3 weeks); 9 sessions. Int: 40% 1RM, to volitional failure. Type: Single-leg knee extension and flexion (OKC). Vol: 4 × failure. Rest: 45 s (sets 1,3,5, cuff on); 90 s (sets 2,4, cuff off). Prog: N/S.	Device: Double-chamber pneumatic cuff (13.5 cm, asymmetric). Pressure: BFR-LL 150 mmHg; Sham-BFR 20 mmHg (not adjusted in real time). LOP: Arbitrary.	Isokinetic KE/KF: Peak torque (Nm); hamstring/quadriceps ratio (%); fatigue index (%). MRI: CSA of KE and KF (mm^2^). Biopsy: mid-portion of vastus lateralis and semitendinosus; analysis of gene expression related to muscle growth, inflammation, and angiogenesis.	Isokinetic strength knee extensors (wk 3 POST-OP): Peak torque 60°/s: #,* IG ↑12%; # CG ↓1%; Interaction *p* = 0.018. Total work 60°/s: #,* IG ↑12%; # CG = 0%; Interaction *p* = 0.038. Peak torque 120°/s: #,* IG ↑9%; # CG ↓3%; Interaction *p* = 0.027. Total work 120°/s: #,* IG ↑8%; # CG ↓2%; Interaction *p* = 0.008. Isokinetic strength knee flexors (wk 3 POST-OP): Total work 60°/s: #,* IG ↑12%; * CG ↑1%; Interaction *p* = 0.026. Total work 120°/s: * IG ↑10%; #,* CG ↓5%; Interaction *p* = 0.030. Fatigue indexes knee extensors (wk 3 POST-OP): Peak torque 60°/s: #,* IG ↓60%; # CG ↓21%; Interaction *p* = 0.008. Total work 60°/s: #,* IG ↓54%; # CG ↓6%; Interaction *p* = 0.002. Peak torque 120°/s: #,* IG ↓52%; # CG ↓6%; Interaction *p* = 0.028. Total work 120°/s: #,* IG ↓45%; # CG ↑3%; Interaction *p* = 0.004. CSA knee extensors (wk 3 POST-OP): #,* IG ↑5%; # CG ↓1%; Interaction *p* = 0.018. Summary: BFR-LL ↑ hypertrophy, cross-sectional area, and quadriceps endurance; hamstrings less responsive. Specific physiological adaptation pattern in knee extensors and flexors detected.
**Tramer et al.** [46] **United States** **2022** **RCT**	PRE-OP Start: fourteen days PRE-OP. End: N/S.	Freq: 5/week (2 weeks); 10 sessions. Int: Body weight and light loads. Type: Quadriceps contractions, straight leg raises, long-arc quads, quarter squats (OKC & CKC). Vol: 4 × 30-15-15-failure. Rest: 30 s (no reperfusion); 2 min deflation between exercises. Prog: Load increased near failure.	Device: Single-chamber pneumatic tourniquet (size N/S). Pressure: BFR-LL 80% LOP (not adjusted in real time). LOP: Calculated via Doppler ultrasound (dorsalis pedis).	Isometric KE: Peak, average, and time-to-peak force (N, s). VAS: Pain. PROMIS: Physical function, pain, and depression. Knee ROM: Degrees.	Summary: Home-based exercise ↑ quadriceps peak force in both groups (no sig. differences). PRE-OP home-based BFR-LL protocol was feasible, accessible, and well tolerated.
**Okoroha et al.** [38] **United States** **2023** **RCT**	PRE-OP Start: fourteen days PRE-OP. End: N/S.	Freq: 5/week (2 weeks); 10 sessions. Int: N/S. Type: Quadriceps contractions, straight leg raises, long-arc quads, quarter squats (OKC & CKC). Vol: 4 × 30-15-15-15. Rest: 30 s (no reperfusion); 2 min deflation between exercises. Prog: N/S.	Device: Single-chamber pneumatic tourniquet (size N/S). Pressure: BFR-LL 80% LOP (not adjusted in real time). LOP: Calculated via Doppler ultrasound (dorsalis pedis).	Quadriceps circumference: Muscle volume (cm). Isometric KE: Peak torque and mean torque (Nm); time-to-peak torque (s). VAS: Pain. IKDC/KOOS: Knee function and symptoms. PROMIS: Physical function, pain, and depression. Knee ROM: Degrees.	Quadriceps index (wk 6 POST-OP): Mean force: # IG ↑57%; CG ↑40%; Statistically significant difference between groups (*p* = 0.029, d = 0.80). Summary: Incorporating BFR-LL into home-based PRE-OP exercise for ACLR → ↑ strength and improved patient-reported outcomes at 6 wk POST-OP. No significant differences at 3 and 6 mo between groups. Suggests perioperative BFR-LL may enhance early POST-OP quadriceps recovery.
**Iversern et al.** [44] **China** **2015** **RCT**	POST-OP Start: two days POST-OP. End: two weeks after start.	Freq: 2/day, 7/week (2 weeks); 28 sessions. Int: N/S. Type: Isometric quadriceps contractions, leg extensions over a roll, straight leg raises (OKC). Vol: 5 × 20 isometric contractions per occlusion. Rest: 3 min (cuff off). Prog: Exercise type progressed (isometric → dynamic).	Device: Pneumatic occlusion cuff (14 cm, contoured). Pressure: 130–180 mmHg, increasing 10 mmHg every 2 days (not adjusted in real time). LOP: Arbitrary.	MRI: CSA of KE (cm^2^).	KE CSA: CSA 40% (PRE-OP vs. POST-OP): # IG ↓13%; # CG ↓12%. CSA 50% (PRE-OP vs. POST-OP): # IG ↓11%; # CG ↓14%. Summary: Home-based BFR-LL rehabilitation intervention → no significant effect on quadriceps CSA (MRI) in athletes.
**Hughes et al. (2019a)** [40] **England** **2019** **RCT**	POST-OP Start: BFR-LL: 23 ± 2 days POST-OP. HL-RT: 24 ± 1 days POST-OP. End: N/S.	Freq: 2/week (8 weeks); 16 sessions. Int: BFR-LL 30% 1RM; HL-RT 70% 1RM. Type: Single-leg press (CKC). Vol: BFR 4 × 30-15-15-15; HL 3 × 10. Rest: 30 s (no reperfusion). Prog: Load increased 10% after 2 successful sessions.	Device: Automatic personalized tourniquet system (11.5 × 86 cm, 5 mm thick, variable contour nylon cuff). Pressure: 80% LOP (auto-regulated in real time). LOP: Calculated by automatic device.	Borg scale: Session, 24h post-training, and muscle pain, perceived exertion (0–11).	Session knee joint pain IL (wk 8 POST-OP): * IG ↓92%; * CG ↓60%. 24 h knee joint pain IL (wk 8 POST-OP): #* IG ↓100%; #* CG ↓91%. Session muscle pain IL (wk 8 POST-OP): * IG ↓31%; * CG ↓47%. Session muscle pain in IL (wk 8 POST-OP): * IG ↓26%; * CG ↓26%. Summary: BFR-LL ↓ knee pain during and 24 h after sessions vs. HL-RT. ↑ muscle pain in both limbs, but without affecting adherence. RPE similar between groups and unchanged during training.
**Hughes et al. (2019)** [41] **England** **2019** **RCT**	POST-OP Start: BFR-LL: 23 ± 2 days POST-OP. HL-RT: 24 ± 1 days POST-OP. End: N/S.	Freq: 2/week (8 weeks); 16 sessions. Int: BFR-LL 30% 1RM; HL-RT 70% 1RM. Type: Single-leg press (CKC). Vol: BFR 4×30-15-15-15; HL 3 × 10. Rest: 30 s (no reperfusion). Prog: Load increased 10% after mid-program test.	Device: Automatic personalized tourniquet system (11.5 × 86 cm, 5 mm thick, variable contour nylon cuff). Pressure: 80% LOP (auto-regulated in real time). LOP: Calculated by automatic device.	Isokinetic KE/KF: Strength (kg/kg·bm). Ultrasound (VL): Muscle thickness (cm), pennation angle (°), fascicle length (cm). IKDC/KOOS/LEFS: Knee and lower-limb function. KOOS-Pain: Pain (0–100). Modified star excursion: Dynamic balance (cm). Knee ROM: Degrees. Effusion: Mid-patella circumference (cm). KT-1000: Laxity (mm).	Summary: Both groups ↑ skeletal muscle hypertrophy and strength similarly. BFR-LL ↓ knee joint pain and effusion → greater overall improvement in functional outcomes.
**Curran et al.** [43] **United States** **2020** **RCT**	POST-OP Start: ten weeks POST-OP. End: N/S.	Freq: 2/week (8 weeks); 16 sessions. Int: Concentric BFR: C70%E20%; Eccentric BFR: C20%E70%. Type: Single-leg press (CKC). Vol: 4 × 10. Rest: 2 min (cuff off). Prog: Weekly load adjustment based on 1RM.	Device: Automatic personalized tourniquet system (11.5 × 86 cm, 5 mm thick, variable contour nylon cuff). Pressure: CBFR-RT and EBFR-RT at 80% LOP. LOP: Calculated by automatic device.	Isometric KE: MVIC (Nm). Isokinetic KE: Strength (Nm); pre-to-post change. EMG (superimposed burst): Quadriceps activation (%). Ultrasound: Rectus femoris muscle volume (cm^3^). IKDC: Knee function and symptoms.	Summary: BFR-HL did not significantly improve the recovery of quadriceps muscle strength, activation, or atrophy compared to HL-RT.
**Alavi et al.** [52] **Iran** **2021** **RCT**	POST-OP Start: three months POST-OP. End: twelve weeks after start.	Freq: 2/week (12 weeks); 24 sessions. Int: BFR-LL and RT 30–70% 1RM. Type: Multi-exercise rehabilitation (OKC & CKC: squats, step-ups, adduction, etc.). Vol: 2–4 × 10 RM. Rest: N/S. Prog: N/S.	Device: N/S. Pressure: 120–180 mmHg. LOP: N/S.	Serum biomarkers: Atrogin-1, MuRF1.	Serum Atrogin-1 levels (POST-OP week 0→12): #,¥ IG: ↓ −12.53% Serum MuRF1 levels (POST-OP week 0→12): ¥ IG: ↓ −15.47% Summary: BFR training ↓ circulating Atrogin-1 and MuRF1—two key proteins involved in muscle proteolysis—indicating a potential inhibitory effect on postoperative muscle atrophy and a positive influence on muscle mass preservation following ACLR.
**Jack et al.** [42] **United States** **2022** **RCT**	POST-OP Start: seven days POST-OP. End: twelve weeks after start.	Freq: 2/week (12 weeks); 24 sessions. Int: 30% 1RM. Type: Rehabilitation exercises (OKC & CKC). Vol: 4 × 30-15-15-15. Rest: 30 s (no reperfusion). Prog: Weekly exercise progression.	Device: Automatic personalized tourniquet system (11.5 × 86 cm, 5 mm thick, variable contour nylon cuff). Pressure: 80% LOP (auto-regulated in real time). LOP: Calculated by automatic device.	DEXA: Bone mineral density (g/cm^2^), bone and lean mass. Single-leg tests: Squat, leg press, hamstring curl (1RM, kg); eccentric step-down (reps to fatigue). Y-balance: Neuromuscular control (cm).	SL squat (week 8→12 POST−OP): * IG: ↑ 29%; * CG: ↑ 19% SL step−down: * IG: ↑ 39%; * CG: ↑ 38% SL press: * IG: ↑ 23%; * CG: ↑ 31% SL hamstring curl: * IG: ↑ 34%; * CG: ↑ 29% Anterior Y−balance: * IG: ↑ 9% Posteromedial Y−balance: * IG: ↑ 7%; * CG: ↑ 12% Posterolateral Y−balance: * IG: ↑ 9%; * CG: ↑ 16% Bone mass (whole limb): Week 6: # IG: = 0%; #,* CG: ↓ −2% Week 12: # IG: ↓ −1%; #,* CG: ↓ −3% Bone mass (femur): Week 6: * CG: ↓ −3% Week 12: #,* IG: ↓ −2%; #,* CG: ↓ −3% Site−specific BMD: Distal femur week 6: # IG: ↓ −3%; #,* CG: ↓ −8% Distal femur week 12: * IG: ↓ −5%; * CG: ↓ −8% Proximal tibia week 6: * CG: ↓ −5% Proximal tibia week 12: # IG: ↓ −2%; #,* CG: ↓ −8% Proximal fibula week 6: * IG: ↓ −7%; * CG: ↓ −13% Proximal fibula week 12: #,* IG: ↓ −7%; #,* CG: ↓ −15% Lean mass (whole limb): Week 6: # IG: ↓ −1%; #,* CG: ↓ −7% Week 12: # IG: ↑ 1%; #,* CG: ↓ −5% Lean mass (thigh): Week 6: * IG: ↓ −3%; #,* CG: ↓ −8% Week 12: * IG: ↑ 0%; #,* CG: ↓ −4% Summary: BFR−LL ↓ muscle and bone loss up to 12 weeks POST−OP and reduced RTS time vs. control group.
**Vieira et al.** [45] **Brasil** **2022** **RCT**	POST-OP Start: hospital discharge. End: twelve weeks after start.	Freq: 2/week (12 weeks); 24 sessions. Int: BFR-LL 30% 1RM; HL-RT 70% 1RM. Type: Leg press and flexor chair (OKC & CKC). Vol: BFR 4 × 30-15-15-15; HL 3 × 10. Rest: 30 s (no reperfusion); 5 min between exercises. Prog: Load increased 10% if all reps completed (weeks 4–12).	Device: Pneumatic cuff (10 × 80 cm). Pressure: BFR-LL 80% LOP (not adjusted in real time). LOP: Calculated via Doppler ultrasound (posterior tibial artery).	Isometric KE/KF: MVIC (Nm). Lysholm/IKDC/KOOS: Knee function and symptoms.	KE muscle strength (injured leg, week 12 POST−OP): * IG: ↑ 11%; * CG: ↓ −22%; *p* < 0.01, d = 1.9 KF muscle strength (injured leg, week 8 POST−OP): * IG: ↑ 5%; * CG: ↓ −25%; *p* < 0.01, d = 1.7 KF muscle strength (injured leg, week 12 POST−OP): * IG: ↑ 28%; * CG: ↓ −16%; *p* < 0.01, d = 3.4 Lysholm scale (week 8 POST−OP): # IG: ↑ 17%; * CG: ↑ 6% Lysholm scale (week 12 POST−OP): # IG: ↑ 17%; *p* = 0.001, d = 4.31 KOOS symptoms (weeks 4–12 POST−OP): * IG: ↑ 4% → #; IG: ↑ 13%; *p* < 0.01, d = 3.53 KOOS pain (weeks 4–12 POST−OP): * IG: ↑ 7% → #;* IG: ↑ 18%; * CG: ↓ −17% → ↓ −9%; *p* < 0.01, d = 1.96 KOOS daily activity (weeks 4–12 POST−OP): * IG: ↑ 4% → #;* IG: ↑ 20% *p* < 0.01, d = 2.38 KOOS quality of life (weeks 4–12 POST−OP): * IG: ↑ 13% → ↑ 30%; *p* < 0.01, d = 1.72 IKDC scale (weeks 4–12 POST−OP): * CG: ↓ −11% → #;* IG: ↑ 25%; *p* < 0.01, d = 3.23 Summary: BFR−LL ↑ quadriceps and hamstring strength and improved functional scores (Lysholm, KOOS, IKDC) more rapidly than control.
**Khalil et al.** [39] **United States** **2023** **RCT**	POST-OP Start: seven days POST-OP. End: until the end of the third postoperative month.	Freq: 3/week (11 weeks); 33 sessions. Int: BFR-LL 30% 1RM; HL-RT 70% 1RM. Type: Rehabilitation plus neuromuscular electrical stimulation, divided into early (CKC) and late (OKC & CKC) phases. Vol: 4 × 30-15-15-15. Rest: N/S. Prog: Phase-based exercise progression (postoperative timeline).	Device: N/S. Pressure: BFR-LL 80% LOP (not adjusted in real time). LOP: Calculated via Doppler ultrasound (site N/S).	VAS: Pain (0–100).	Summary: A conventional rehabilitation program ↓ knee pain post−ACLR. Adding BFR−LL to standard rehab showed no additional effect on pain reduction compared with conventional therapy alone.
**Sevinc et al.** [50] **Turkey** **2024** **RCT**	POST-OP Start: four weeks POST-OP. End: twelve weeks POST-OP.	Freq: 2/week (8 weeks); 16 sessions. Int: BFR + cross-education N/S; cross-education 7–8 RPE. Type: Isokinetic cross-education (uninjured limb) plus standard rehabilitation (weeks 0–12). Vol: 3 × 12 at 60°/s (10–90° flexion). Rest: 2 min between sets. Prog: N/A.	Device: Pneumatic cuff (5 cm width). Pressure: Continuous BFR + cross-education 80% LOP (not adjusted in real time). LOP: Calculated via Doppler ultrasound (posterior tibial artery, seated).	Isometric KE: MVIC (Nm). Ultrasound: Muscle thickness (mm) and CSA (cm^2^) of quadriceps.	Summary: Adding BFR to cross−education training did not confer additional benefits in strength recovery after ACLR.
**Erickson et al.** [53] **United States** **2024** **RCT**	PRE- and POST-OP Start: one month PRE-OP. End: four months of therapy if no weightbearing restrictions, five months if restrictions due to delayed loading.	Freq: 3/week (24 weeks); 72 sessions. Int: BFR-LL 20–30% 1RM; Sham-BFR 60–70% 1RM; failure by last set. Type: Pre- and postoperative rehabilitation (leg press, knee extension, step-ups, squats, straight leg raises). Vol: BFR 3 × 30-20-10; Sham-BFR 3 × 10. Rest: 30 s between sets; 1–2 min between exercises. Prog: Load, volume, or intensity increased if RPE <7.	Device: Automatic personalized tourniquet system (Easi-Fit cuff) for BFR-LL; non-automatic bands for Sham-BFR. Pressure: BFR-LL 60% occlusion (auto-regulated); Sham-BFR 20 mmHg. LOP: Calculated automatically.	Quadriceps strength: Isometric/isokinetic peak torque and rate of torque development. Morphology: CSA, pennation angle, fiber length, and volume. Knee biomechanics: Extensor moment and flexion angle. Estimation of muscle fibrosis: T1rho Measurement. Muscle biopsy: Fiber type, CSA, satellite cell content, collagen, fibro/adipogenic progenitor cells.	BFR−RT and Sham−BFR produced similar improvements in quadriceps muscle function post−ACLR.

Symbols: # Significant Interaction group × time, *p* < 0.05; * Significant effect of group, *p* < 0.05; ¥ Significant effect of Time, *p* < 0.05; ↑ indicates an increase; ↓ indicates a decrease; = indicates no change. Abbreviations: ACL, anterior cruciate ligament; ACLR, anterior cruciate ligament reconstruction; BFR, blood flow restriction; BFR-LL, Blood flow restriction light load; BFR-RT, blood flow restriction resistance training; BFR-ET, blood flow restriction endurance training; BFR-HL, blood flow restriction heavy load; CBFR-RT, concentric blood flow restriction resistance training; EBFR-RT, eccentric blood flow restriction resistance training; BMD, bone mineral density; CSA, cross-sectional area; CKC, closed kinetic chain; OKC, open kinetic chain; DEXA, Dual-energy-X-ray-absorptiometry; EBFR-HL, eccentric blood flow restriction with heavy load; FI, Fatigue indexes; FITT-VP, frequency, intensity, type, time, volume, and progression; HL-RT, high load resistance training; IG, intervention group; IKDC, International knee documentation Committee; IL, injured leg; KF, knee flexion; KE, knee extension; KOOS, Knee Injury and Osteoarthritis Outcome Score; LEFS, lower extremity functional scale; LOP, limb occlusive pressure; MRI, magnetic resonance imaging; MVIC, Maximum voluntary isometric contraction; NIRS, near-infrared spectroscopy; N/S, not specified; PROMIS, Patient-Reported Outcomes Measurement Information System; RMS, root mean square; RTS, return to sport; POST, posterior; POST-OP, postoperative; PRE-OP, preoperative; RPE, Rate of perceived exertion; VAS, visual analogic scale. ^a^ Time of intervention, author, country, year, design.

**Table 4:** Identified knowledge gaps in the literature and recommendations for future studies.

Identified Knowledge Gaps from Scoping Review	Implications for Future Research
Lack of comparative studies and limited evidence	Few studies directly compare BFR-RT with other exercise modalities or control groups, limiting our understanding of its relative effects and outcomes across different populations and contexts.
Lack of studies with diverse in participant populations	Most BFR-RT and ACLR studies have included primarily young, healthy participants without additional pathologies. Future research should focus on specific sports, age groups, and surgical interventions to increase generalizability.
Lack of understanding of the physiological mechanisms	The physiological mechanisms underlying BFR-RT adaptations in ACLR recovery remain unclear. Further studies are needed to elucidate the specific responses and adaptations, providing deeper insight into how BFR-RT influences healing and recovery.
Lack of research on long-term effects	Longitudinal studies on prolonged BFR-RT effects, specifically examining the long-term effects on muscle function, physical and physiological parameters, as well as potential adverse effects, are limited. More research is needed to understand the long-term effects of BFR-RT interventions.
Lack of standardized protocols	The absence of consensus on standardized BFR-RT protocols and parameters challenges the translation of findings. Establishing evidence-based guidelines for optimal BFR-RT protocols is crucial for enhancing consistency and facilitating effective implementation of BFR-RT.
Lack of comparative studies and/or meta-analyses examining the dosage effects.	There is a need for comparative studies and/or future meta-analyses to elucidate the dosage effects of BFR-RT. This would help determine the optimal BFR-RT protocols and dosages for different populations and outcomes.
Lack of standardized scales for assessing the methodological description.	Scales are needed to accurately describe and compare key BFR-RT parameters, enhancing research transparency and reproducibility.
Lack of studies on the PRE-OP of BFR-RT application in ACLR:	There is a lack of studies investigating the effects of PRE-OP BFR-RT application as part of rehabilitation following an ACL injury.
Lack of standardized BFR devices	There is a need for studies that compare different BFR devices, as currently, no device has been validated their methods for calculating the Limb Occlusion Pressure (LOP).
Feasibility of blinding and use of sham conditions	Research must focus on developing and validating effective sham/placebo BFR conditions. Studies should also rigorously document and justify the blinding strategy (or lack thereof) to enhance internal validity.

## Data Availability

Not applicable.

## Supplementary Materials

The following supporting information can be downloaded at: https://www.mdpi.com/article/10.3390/jfmk10040450/s1, PRISMA 2020 Checklist.

## Abbreviations

The following abbreviations are used in this manuscript:

ACL	Anterior cruciate ligament
ACLR	Anterior cruciate ligament reconstruction
BC	Body composition
BFR	Blood flow restriction
BFR-LL	Blood flow restriction light load
BFR-RT	Blood flow restriction resistance training
BMD	Bone mineral density
BMI	Body mass index
BTB	Bone-patella tendon-bone autograft
CBFR-HL	Concentric blood flow restriction with heavy load
CG	Comparison group
CKC	Closed kinetic chain
CSA	Cross-sectional area
DEXA	Dual-energy X-ray absorptiometry
EBFR-HL	Eccentric blood flow restriction with heavy load
FI	Fatigue indexes
FITT-VP	Frequency, intensity, type, time, volume, and progression
FM	Functional measurements
HL-RT	High load resistance training
HS	Hamstrings tendon
IG	Intervention group
IKDC	International Knee Documentation Committee
IL	Injured leg
KE	Knee extension
KOOS	Knee Injury and Osteoarthritis Outcome Score
LEFS	Lower extremity functional scale
LOP	Limb occlusive pressure
M	Man
MMHG	Millimeters of mercury
MRI	Magnetic resonance imaging
MPB	Muscle physiology and biomarkers
MVIC	Maximum voluntary isometric contraction
N	Sample number
NMAR	Neuromuscular adaptations and responses
N/S	Not specified
OKC	Open kinetic chain
POST	Posterior
POST-OP	Postoperative
PRE-OP	Preoperative
QRCT	Quasi-randomized controlled trial
QT	Quadriceps tendon
RCT	Randomized controlled trial
RM	Repetition maximum
RPE	Rate of perceived exertion
RTA	Return to activity
RT	Resistance training
SRQ	Self-report questionnaires
VAS	Visual analog scale
W	Woman
y.o	Years old

## Appendix A

**Table A1:** Electronic Strategy of search.

Database	Search Fields	Search Terms	Results
Web of Science	Topic (Title, abstract, key words)	(TS = (“vascular occlu *” OR “blood flow restrict *” OR “blood flow occlu *” OR kaatsu OR “partial occlu *”)) AND (TS = (“anterior cruciate ligament reconstruction” OR “ACLR” OR “anterior cruciate ligament surgery” OR “ACL surgery” OR “anterior cruciate ligament”)).	89
PEDro	Abstract & Title	blood flow restriction * AND anterior cruciate ligament *	22
Scopus	Title, abstract, key words	(TITLE-ABS-KEY(“vascular occlu *” OR “blood flow restrict *” OR “blood flow occlu *” OR “kaatsu” OR “partial occlu *” ) AND TITLE-ABS-KEY (“anterior cruciate ligament reconstruction” OR “ACLR” OR “anterior cruciate ligament surgery” OR “ACL surgery” OR “anterior cruciate ligament”))	112
PUBMED	Title, abstract, key words	(TITLE-ABS-KEY = (“vascular occlu *” OR “blood flow restrict *” OR “blood flow occlu *” OR kaatsu OR “partial occlu *”)) AND (TITLE-ABS-KEY(“anterior cruciate ligament reconstruction” OR “ACLR” OR “anterior cruciate ligament surgery” OR “ACL surgery” OR “anterior cruciate ligament”))	74
SPORTDiscuss	Subject terms	(vascular occlu * OR blood flow restrict * OR blood flow occlu * OR kaatsu OR partial occlu *) AND (anterior cruciate ligament reconstruction OR ACLR OR anterior cruciate ligament surgery OR ACL surgery OR anterior cruciate ligament)	61
Cochrane	Title, abstract, key words	(vascular occlu * OR blood flow restrict * OR blood flow occlu * OR kaatsu OR partial occlu *) AND (anterior cruciate ligament reconstruction OR ACLR OR anterior cruciate ligament surgery OR ACL surgery OR anterior cruciate ligament)	83

*: The asterisks act as truncation symbols. Their purpose is to instruct the search database to retrieve all possible endings or variations of the word root.

## Appendix B

**Table A2:** Assessment of Physiotherapy Evidence Database(PEDro) [36].

		PEDro Scale											
	Study	1	2	3	4	5	6	7	8	9	10	11	Total (0/10)
PRE-OP	Žargi et al. (2016) [49]	Y	Y	Y	Y	N	Y	Y	Y	N	Y	Y	8
	Žargi et al. (2018) [48]	Y	Y	Y	Y	N	Y	N	Y	N	Y	Y	7
	Kacin et al. (2021) [47]	Y	N	N	Y	Y	N	Y	Y	Y	Y	Y	7
	Tramer et al. (2022) [46]	Y	Y	N	Y	N	N	Y	Y	Y	Y	Y	7
	Okoroha et al. (2023) [38]	N	Y	Y	Y	N	N	N	N	Y	Y	Y	6
POST-OP	Iversern et al. (2015) [44]	N	Y	N	Y	N	N	Y	Y	N	Y	Y	6
	Hughes et al. (2019a) [40]	N	Y	Y	Y	N	N	Y	Y	N	Y	Y	7
	Hughes et al. (2019b) [41]	N	Y	Y	Y	N	N	Y	Y	N	Y	Y	7
	Curran et al. (2020) [43]	Y	Y	Y	Y	N	N	N	Y	N	Y	Y	6
	Alavi et al. (2020) [52]	Y	Y	N	Y	N	N	N	Y	Y	Y	Y	7
	Jack et al. (2022) [42]	N	Y	N	Y	N	N	Y	Y	N	Y	Y	6
	Vieira et al. (2022) [45]	Y	Y	Y	Y	N	N	N	Y	Y	Y	Y	7
	Khalil et al. (2023) [39]	N	Y	N	Y	Y	N	Y	Y	N	Y	Y	7
	Sevinc et al. (2024) [50]	Y	Y	N	Y	N	N	Y	Y	N	Y	Y	6
PRE- and POST-OP	Erickson et al. (2024) [53]	Y	Y	Y	Y	Y	N	Y	Y	Y	Y	Y	9

1-Eligibility criteria(No value); 2-Specified random allocation; 3-Concealed allocation; 4-Groups similar at baseline; 5-Subject blinding; 6-Therapist blinding; 7-Assessor blinding; 8-Measures of one key outcome(85%); 9-Intention-to-treat analysis; 10-Between group statistical comparisons; 11-Point measures and variability data; Y, Yes; N, No.

## Appendix C

**Table A3:** Assessment of Consensus on Exercise Reporting Template(CERT) [37].

	Study	1	2	3	4	5	6	7a	7b	8	9	10	11	12	13	14a	14b	15	16a	16b	Overall
PRE-OP	Žargi et al. (2016) [49]	Y	Y	N	Y	N	N	N	N	Y	Y	N	N	Y	Y	Y	N	Y	N	Y	10
	Žargi et al. (2018) [48]	N	Y	Y	Y	N	N	N	N	Y	Y	N	N	Y	Y	Y	N	Y	N	N	9
	Kacin et al. (2021) [47]	Y	N	N	Y	N	N	N	N	Y	Y	N	N	Y	Y	Y	N	Y	N	N	8
	Joseph et al. (2022) [46]	Y	N	Y	Y	N	N	N	Y	Y	Y	N	Y	Y	Y	Y	N	Y	N	N	10
	Okoroha et al. (2023) [38]	Y	Y	Y	Y	N	N	N	N	Y	Y	N	Y	Y	Y	Y	N	Y	N	N	12
	TOTAL(%) [46,47,48,49]	80	60	60	100	0	0	0	20	100	100	0	40	100	100	100	0	100	0	20	51.6%
POST-OP	Iversern et al. (2015) [44]	Y	N	Y	Y	N	N	Y	Y	Y	Y	N	N	Y	Y	Y	N	Y	N	N	11
	Hughes et al. (2019a) [40]	Y	Y	N	Y	Y	N	Y	Y	Y	Y	Y	Y	Y	Y	Y	Y	Y	Y	Y	17
	Hughes et al. (2019b) [41]	Y	Y	N	Y	Y	N	Y	Y	Y	Y	Y	Y	Y	Y	Y	Y	Y	Y	Y	17
	Curran et al. (2020) [43]	Y	N	N	Y	N	N	Y	Y	Y	N	N	N	Y	Y	Y	Y	Y	Y	N	11
	Alavi et al. (2020) [52]	N	N	N	Y	N	N	N	N	Y	N	N	N	Y	Y	Y	Y	Y	N	N	7
	Jack et al. (2022) [42]	Y	Y	N	Y	N	N	N	Y	Y	N	N	Y	Y	Y	Y	Y	Y	N	N	11
	Vieira et al. (2022) [45]	Y	N	N	Y	Y	N	Y	Y	Y	N	N	Y	Y	Y	Y	Y	Y	Y	N	13
	Khalil et al. (2023) [39]	N	N	N	N	N	N	N	Y	N	Y	N	N	Y	Y	Y	N	Y	N	N	6
	Sevinc et al. (2024) [50]	Y	Y	N	N	N	N	N	Y	Y	Y	N	Y	Y	Y	Y	Y	Y	N	N	11
	TOTAL (%) [40,41,42,43,44,45,50]	77.8	44.4	11.1	77.8	33.3	0	55.6	88.9	88.9	55.6	22.2	55.6	100	100	100	77.8	100	42.9	22.2	60.8%
PRE- and POST-OP	Erickson et al. (2024) [53]	Y	Y	Y	Y	N	N	Y	Y	Y	N	Y	N	Y	Y	Y	Y	Y	N	N	13

1-Detailed description of the type of exercise equipment; 2-Detailed description of the qualifications, expertise and/or training; 3-Describe whether exercises are performed individually or in a group; 4-Describe whether exercises are supervised or unsupervised; how they are delivered; 5-Detailed description of how adherence to exercise is measured and reported; 6-Detailed description of motivation strategies; 7a-Detailed description of the decision rule(s) for determining exercise progression; 7b-Detailed description of how the exercise program was progressed; 8-Detailed description of each exercise to enable replication; 9-Detailed description of any home program component; 10-Describe whether there are any no-exercise components; 11-Describe the type and number of adverse events that occur during exercise; 12-Describe the setting in which the exercises are performed; 13-Detailed description of the exercise intervention; 14a-Describe whether the exercises are generic (one size fits all) or tailored; 14b-Detailed description of how exercises are tailored to the individual; 15-Describe the decision rule for determining the starting level; 16a-Describe how adherence or fidelity is assessed/measured; 16b-Describe the extent to which the intervention was delivered as planned; Y, Yes; N, No.

## Appendix E

**Table A4:** Tier List of Clinical Outcomes: Efficacy of BFR Training in ACLR.

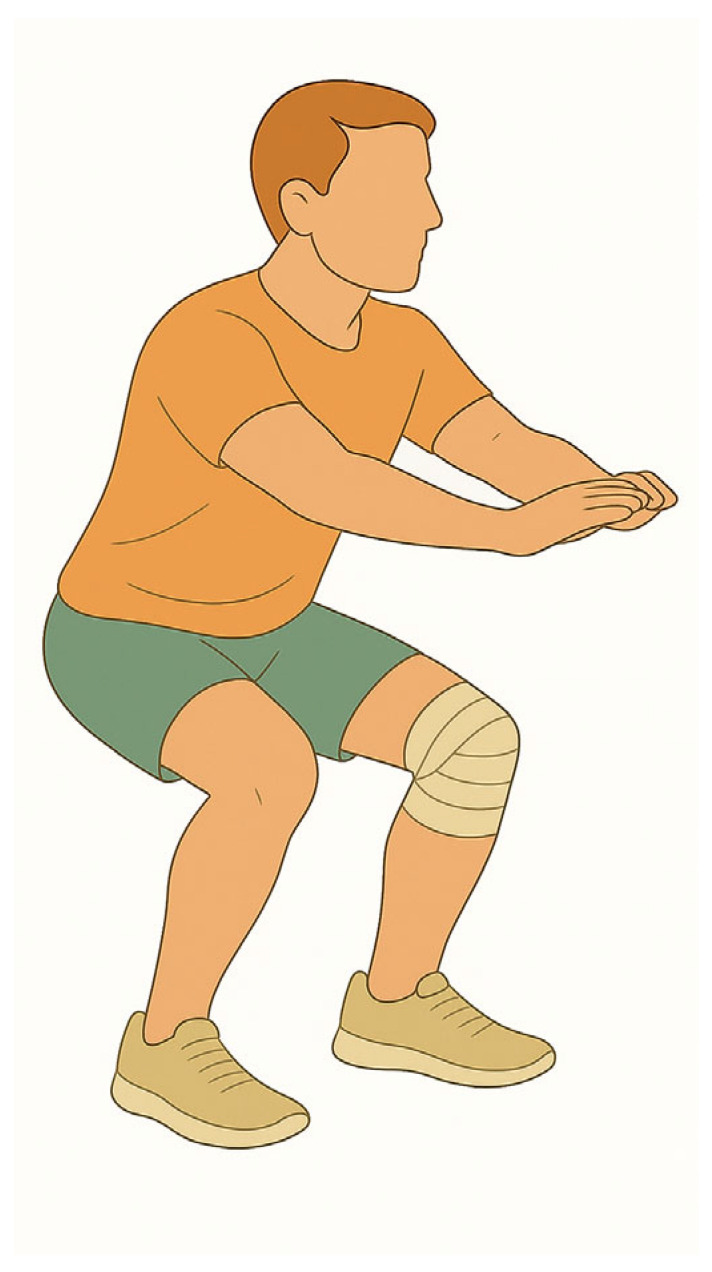
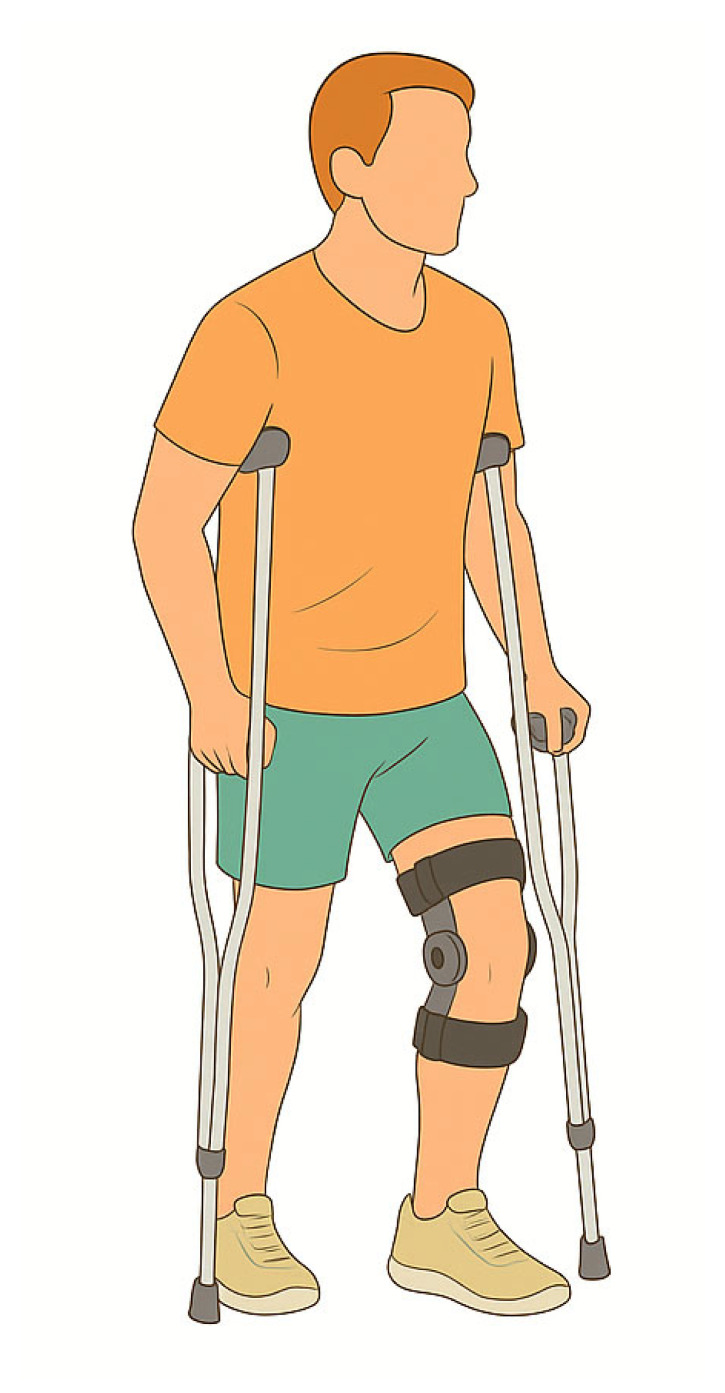
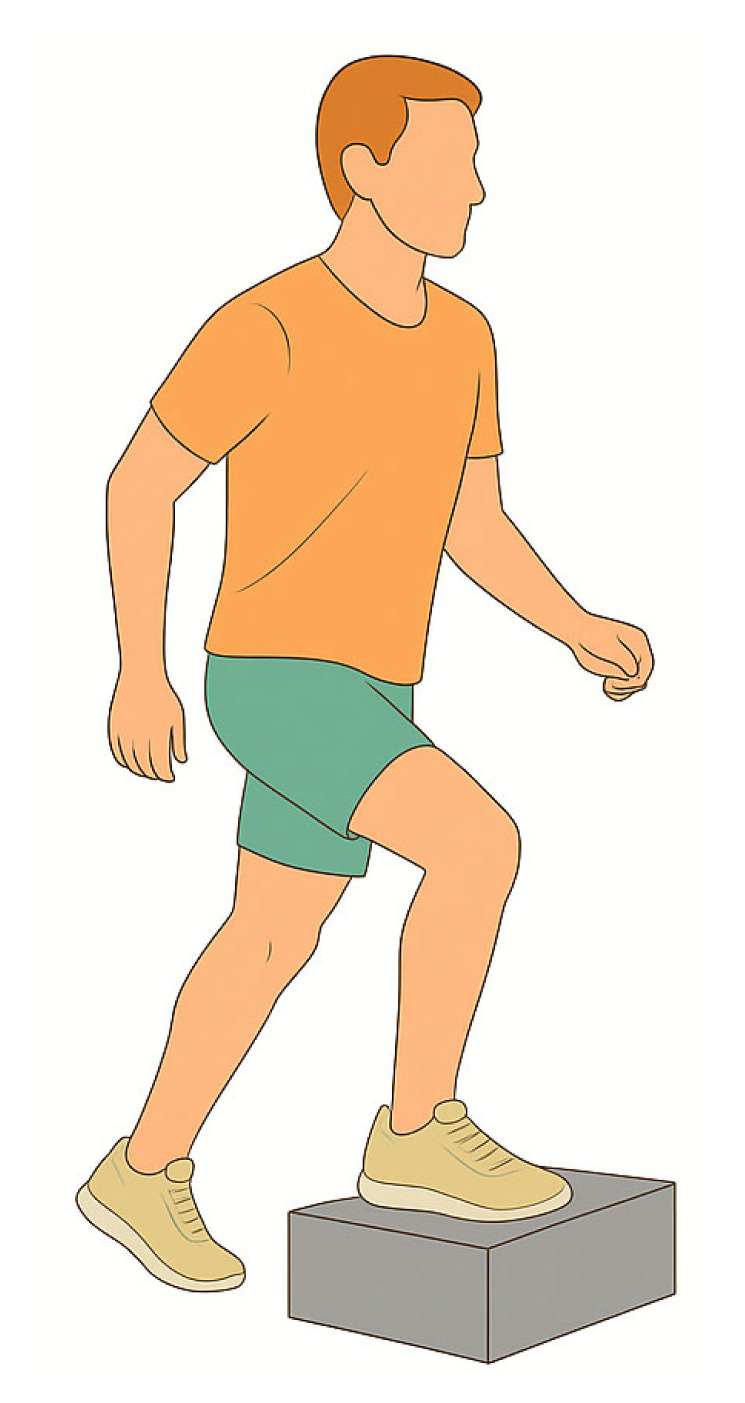

			
Outcome/Phase	PRE-OP	Early POST-OP (0–2 m)	Medium POST-OP (>2 m)
**Body Composition**	BFR-RT ≥ CSA LL-RT/Sham-BFR (B)	BFR-LL = CSA vs. HL-RT (A) BFR-LL + home-based isometric protocol = Strength/CSA vs. Sham-BFR (C)	BFR-LL = CSA vs. HL-RT (A) BFR-LL ↑ bone mass/lean mass vs. LL-RT (A)
**Neuromuscular** **Adaptations**	BFR-LL ↑ Strength vs. LL-RT/Sham-BFR (A)	BFR-LL ≥ knee extensors Strength vs. HL-RT (B)	BFR-LL + cross-education = Strength vs. Sham-BFR (B)
**Self-Reported** **Outcomes**	BFR-LL (home-based PRE-OP) ≥ Strength/CSA vs. LL-RT (B)	BFR-RT ↓Pain vs. HL-RT (S) BFR-LL ↑Pain immediate vs. HL-RT (C)	BFR-LL = Pain/Function vs. LL-RT (B)
**Functional** **Measurements**	BFR-LL = ROM/Y-balance vs. LL-RT/Sham-BFR (C)	BFR-LL ↑Y-balance vs. HL-RT (A) BFR-LL ↑ knee ROM vs. HL-RT (A) BFR-LL = knee laxity vs. HL-RT (A)	BFR-LL ↑ Y-balance vs. LL-RT(A)
**Muscle Physiology and Biomarkers**	BFR-LL ↑ Vascular/VEGF-A vs. Sham-BFR (S)	No reported	BFR-RT ↓ Atrophy biomarkers vs. LL-RT (B)
**Return to Activity Time**	No reported	No reported	BFR-LL ↓ RTS vs. LL-RT (A) BFR-HL = RTS vs. HL-RT (C)

Symbols: ↑ indicates an increase; ↓ indicates a decrease; = indicates no change; ≥ indicates greater than or equal to. The Tier List provides a visual ranking of BFR protocol efficacy based on relative advantage: Tier S (Superiority), significantly better than the comparator; Tier A (High Efficacy), more effective but requiring further evidence; Tier B (Mixed/Protective), mildly positive with some contradictions; Tier C (Ineffective/Redundant), no added benefit when added to an effective protocol. Abbreviations: BFR, blood flow restriction; BFR-HL, BFR + high-load; BFR-LL, BFR + low-load; BFR-RT, BFR resistance training; CSA, cross-sectional area; HL-RT, high-load RT; LL-RT, low-load RT; m, Month; Post-Op, postoperative; Pre-Op, preoperative; ROM, range of motion; RTS, return to sport/activity; Tier A/B/C/S, structured evidence classification; VEGF-A, vascular endothelial growth factor A; Y-balance, Y-Balance Test.

## References

[1] Chia L., De Oliveira Silva D., Whalan M., McKay M.J., Sullivan J., Fuller C.W., Pappas E. (2022). Non-Contact Anterior Cruciate Ligament Injury Epidemiology in Team-Ball Sports: A Systematic Review with Meta-Analysis by Sex, Age, Sport, Participation Level, and Exposure Type. Sports Med..

[2] Weitz F.K., Sillanpää P.J., Mattila V.M. (2020). The incidence of paediatric ACL injury is increasing in Finland. Knee Surg. Sports Traumatol. Arthrosc..

[3] Bram J.T., Magee L.C., Mehta N.N., Patel N.M., Ganley T.J. (2020). Anterior Cruciate Ligament Injury Incidence in Adolescent Athletes: A Systematic Review and Meta-Analysis. Am. J. Sports Med..

[4] Paudel Y.R., Sommerfeldt M., Voaklander D. (2023). Increasing incidence of anterior cruciate ligament reconstruction: A 17-year population-based study. Knee Surg. Sports Traumatol. Arthrosc..

[5] Allahabadi S., Rubenstein W.J., Lansdown D.A., Feeley B.T., Pandya N.K. (2020). Incidence of anterior cruciate ligament graft tears in high-risk populations: An analysis of professional athlete and pediatric populations. Knee.

[6] Hespanhol L.C., Kamper S.J. (2015). Prevention of non-contact anterior cruciate ligament injuries: PEDro synthesis. Br. J. Sports Med..

[7] Eggerding V., Reijman M., Meuffels D.E., Van Es E., Van Arkel E., Van Den Brand I., van Linge J., Zijl J., Bierma-Zeinstra S.M.A., Koopmanschap M. (2022). ACL reconstruction for all is not cost-effective after acute ACL rupture. Br. J. Sports Med..

[8] Stewart B.A., Momaya A.M., Silverstein M.D., Lintner D. (2017). The Cost-Effectiveness of Anterior Cruciate Ligament Reconstruction in Competitive Athletes. Am. J. Sports Med..

[9] Deviandri R., van der Veen H.C., Lubis A.M.T., van den Akker-Scheek I., Postma M.J. (2022). Cost-effectiveness of ACL treatment is dependent on age and activity level: A systematic review. Knee Surg. Sports Traumatol. Arthrosc..

[10] Tim-Yun Ong M., Fu S.C., Mok S.W., Franco-Obregón A., Lok-Sze Yam S., Shu-Hang Yung P. (2022). Persistent quadriceps muscle atrophy after anterior cruciate ligament reconstruction is associated with alterations in exercise-induced myokine production. Asia Pac. J. Sports Med. Arthrosc. Rehabil. Technol..

[11] Baron J.E., Parker E.A., Duchman K.R., Westermann R.W. (2020). Perioperative and Postoperative Factors Influence Quadriceps Atrophy and Strength After ACL Reconstruction: A Systematic Review. Orthop. J. Sports Med..

[12] Øiestad B.E., Juhl C.B., Culvenor A.G., Berg B., Thorlund J.B. (2022). Knee extensor muscle weakness is a risk factor for the development of knee osteoarthritis: An updated systematic review and meta-analysis including 46,819 men and women. Br. J. Sports Med..

[13] Spiering B.A., Clark B.C., Schoenfeld B.J., Foulis S.A., Pasiakos S.M. (2023). Maximizing Strength: The Stimuli and Mediators of Strength Gains and Their Application to Training and Rehabilitation. J. Strength Cond. Res..

[14] Jorgenson K.W., Phillips S.M., Hornberger T.A. (2020). Identifying the Structural Adaptations That Drive the Mechanical Load-Induced Growth of Skeletal Muscle: A Scoping Review. Cells.

[15] Swinton P.A., Schoenfeld B.J., Murphy A. (2024). Dose–Response Modelling of Resistance Exercise Across Outcome Domains in Strength and Conditioning: A Meta-Analysis. Sports Med..

[16] Kotsifaki R., Korakakis V., King E., Barbosa O., Maree D., Pantouveris M., Bjerregaard A., Luomajoki J., Wilhelmsen J., Whiteley R. (2023). Aspetar clinical practice guideline on rehabilitation after anterior cruciate ligament reconstruction. Br. J. Sports Med..

[17] Hill E.C., Rivera P.M., Proppe C.E., Rojas D.H.G., Lawson J.E. (2023). Acute effects of low load blood flow restricted and non restricted exercise on muscle excitation, neuromuscular efficiency, and average torque. J. Musculoskelet. Neuronal Interact..

[18] Olmos A.A., Montgomery T.R., Sears K.N., Dinyer T.K., Hammer S.M., Bergstrom H.C., Hill E.C., Succi P.J., Lawson J., Trevino M.A. (2024). Blood flow restriction increases necessary muscle excitation of the elbow flexors during a single high-load contraction. Eur. J. Appl. Physiol..

[19] Li S., Li S., Wang L., Quan H., Yu W., Li T., Li W. (2022). The Effect of Blood Flow Restriction Exercise on Angiogenesis-Related Factors in Skeletal Muscle Among Healthy Adults: A Systematic Review and Meta-Analysis. Front. Physiol..

[20] Preobrazenski N., Islam H., Gurd B.J. (2021). Molecular regulation of skeletal muscle mitochondrial biogenesis following blood flow-restricted aerobic exercise: A call to action. Eur. J. Appl. Physiol..

[21] Zhao Y.C., Gao B.H. (2024). Integrative effects of resistance training and endurance training on mitochondrial remodeling in skeletal muscle. Eur. J. Appl. Physiol..

[22] De Almeida A.M., Silva P.R.S., Pedrinelli A., Hernandez A.J. (2018). Aerobic fitness in professional soccer players after anterior cruciate ligament reconstruction. PLoS ONE.

[23] Ladlow P., Coppack R.J., Dharm-Datta S., Conway D., Sellon E., Patterson S.D., Bennett A.N. (2018). Low-load resistance training with blood flow restriction improves clinical outcomes in musculoskeletal rehabilitation: A single-blind randomized controlled trial. Front. Physiol..

[24] Wernbom M., Järrebring R., Andreasson M.A., Augustsson J. (2009). Acute effects of blood flow restriction on muscle activity and endurance during fatiguing dynamic knee extensions at low load. J. Strength Cond. Res..

[25] Gopinatth V., Garcia J.R., Reid I.K., Knapik D.M., Verma N.N., Chahla J. (2025). Blood Flow Restriction Enhances Recovery After Anterior Cruciate Ligament Reconstruction: A Systematic Review and Meta-Analysis of Randomized Controlled Trials. Arthroscopy.

[26] Lu Y., Patel B.H., Kym C., Nwachukwu B.U., Beletksy A., Forsythe B., Chahla J. (2020). Perioperative Blood Flow Restriction Rehabilitation in Patients Undergoing ACL Reconstruction: A Systematic Review. Orthop. J. Sports Med..

[27] Koc B.B., Truyens A., Heymans M.J.L.F., Jansen E.J.P., Schotanus M.G.M. (2022). Effect of Low-Load Blood Flow Restriction Training After Anterior Cruciate Ligament Reconstruction: A Systematic Review. Int. J. Sports Phys. Ther..

[28] Telfer S., Calhoun J., Bigham J.J., Mand S., Gellert J.M., Hagen M.S., Kweon C.Y., Gee A.O. (2021). Biomechanical Effects of Blood Flow Restriction Training After ACL Reconstruction. Med. Sci. Sports Exerc..

[29] Peters M.D.J., Marnie C., Tricco A.C., Pollock D., Munn Z., Alexander L., McInerney P., Godfrey C.M., Khalil H. (2020). Updated methodological guidance for the conduct of scoping reviews. JBI Evid. Synth..

[30] Munn Z., Peters M.D.J., Stern C., Tufanaru C., McArthur A., Aromataris E. (2018). Systematic review or scoping review? Guidance for authors when choosing between a systematic or scoping review approach. BMC Med. Res. Methodol..

[31] Tricco A.C., Lillie E., Zarin W., O’Brien K.K., Colquhoun H., Levac D., Moher D., Peters M.D.J., Horsley T., Weeks L. (2018). PRISMA Extension for Scoping Reviews (PRISMA-ScR): Checklist and Explanation. Ann. Intern. Med..

[32] Ardern C.L., Büttner F., Andrade R., Weir A., Ashe M.C., Holden S., Impellizzeri F.M., Delahunt E., Dijkstra H.P., Mathieson S. (2022). Implementing the 27 PRISMA 2020 Statement items for systematic reviews in the sport and exercise medicine, musculoskeletal rehabilitation and sports science fields: The PERSiST (implementing Prisma in Exercise, Rehabilitation, Sport medicine and SporTs science) guidance. Br. J. Sports Med..

[33] Howard B.E., Phillips J., Miller K., Tandon A., Mav D., Shah M.R., Holmgren S., Pelch K.E., Walker V., Rooney A.A. (2016). SWIFT-Review: A text-mining workbench for systematic review. Syst. Rev..

[34] Haddaway N.R., Grainger M.J., Gray C.T. (2022). Citationchaser: A tool for transparent and efficient forward and backward citation chasing in systematic searching. Res. Synth. Methods.

[35] Ouzzani M., Hammady H., Fedorowicz Z., Elmagarmid A. (2016). Rayyan-a web and mobile app for systematic reviews. Syst. Rev..

[36] Maher C.G., Sherrington C., Herbert R.D., Moseley A.M., Elkins M. (2003). Reliability of the PEDro Scale for Rating Quality of Randomized Controlled Trials. Phys. Ther..

[37] Slade S.C., Dionne C.E., Underwood M., Buchbinder R. (2016). Consensus on Exercise Reporting Template (CERT): Explanation and Elaboration Statement. Br. J. Sports Med..

[38] Okoroha K.R., Tramer J.S., Khalil L.S., Jildeh T.R., Abbas M.J., Buckley P.J., Lindell C., Moutzouros V. (2023). Effects of a Perioperative Blood Flow Restriction Therapy Program on Early Quadriceps Strength and Patient-Reported Outcomes After Anterior Cruciate Ligament Reconstruction. Orthop. J. Sports Med..

[39] Khalil A.A., Fayaz N.A., Fawzy E., Mohamed N.A., Waly A.H., Mohammed M.M. (2023). Influence of Blood Flow Restriction Training on Knee Pain After Anterior Cruciate Ligament Reconstruction: A Double Blinded Randomized Controlled Trial. J. Popul. Ther. Clin. Pharmacol..

[40] Hughes L., Patterson S.D., Haddad F., Rosenblatt B., Gissane C., McCarthy D., Clarke T., Ferris G., Dawes J., Paton B. (2019). Examination of the comfort and pain experienced with blood flow restriction training during post-surgery rehabilitation of anterior cruciate ligament reconstruction patients: A UK National Health Service trial. Phys. Ther. Sport.

[41] Hughes L., Rosenblatt B., Haddad F., Gissane C., McCarthy D., Clarke T., Ferris G., Dawes J., Paton B., Patterson S.D. (2019). Comparing the Effectiveness of Blood Flow Restriction and Traditional Heavy Load Resistance Training in the Post-Surgery Rehabilitation of Anterior Cruciate Ligament Reconstruction Patients: A UK National Health Service Randomised Controlled Trial. Sports Med..

[42] Jack R.A., Lambert B.S., Hedt C.A., Delgado D., Goble H., McCulloch P.C. (2022). Blood Flow Restriction Therapy Preserves Lower Extremity Bone and Muscle Mass After ACL Reconstruction. Sports Health.

[43] Curran M.T., Bedi A., Mendias C.L., Wojtys E.M., Kujawa M.V., Palmieri-Smith R.M. (2020). Blood Flow Restriction Training Applied with High-Intensity Exercise Does Not Improve Quadriceps Muscle Function After Anterior Cruciate Ligament Reconstruction: A Randomized Controlled Trial. Am. J. Sports Med..

[44] Iversen E., Røstad V., Larmo A. (2016). Intermittent blood flow restriction does not reduce atrophy following anterior cruciate ligament reconstruction. J. Sport Health Sci..

[45] Vieira de Melo R.F., Komatsu W.R., Freitas M.S.d., Vieira de Melo M.E., Cohen M. (2022). Comparison of Quadriceps and Hamstring Muscle Strength After Exercises with and Without Blood Flow Restriction Following Anterior Cruciate Ligament Surgery: A Randomized Controlled Trial. J. Rehabil. Med..

[46] Tramer J.S., Khalil L.S., Jildeh T.R., Abbas M.J., McGee A., Lau M.J., Moutzouros V., Okoroha K.R. (2022). Blood Flow Restriction Therapy for Two Weeks Prior to Anterior Cruciate Ligament Reconstruction Did Not Impact Quadriceps Strength Compared to Standard Therapy. Arthrosc. J. Arthrosc. Relat. Surg..

[47] Kacin A., Drobnič M., Marš T., Miš K., Petrič M., Weber D., Žargi T.T., Martinčič D., Pirkmajer S. (2021). Functional and molecular adaptations of quadriceps and hamstring muscles to blood flow restricted training in patients with ACL rupture. Scand. J. Med. Sci. Sport..

[48] Žargi T., Drobnič M., Stražar K., Kacin A. (2018). Short-term preconditioning with blood flow restricted exercise preserves quadriceps muscle endurance in patients after anterior cruciate ligament reconstruction. Front. Physiol..

[49] Žargi T.G., Drobnič M., Koder J., Strazar K., Kacin A. (2016). The effects of preconditioning with ischemic exercise on quadriceps femoris muscle atrophy following anterior cruciate ligament reconstruction: A quasi-randomized controlled trial. Eur. J. Phys. Rehabil. Med..

[50] Sevinc C., Gürler V., Harput G., Ocguder A., Ergen F.B., Tunay V.B. (2024). Blood flow restriction training with cross education for quadriceps muscle recovery after anterior cruciate ligament reconstruction: A prospective, randomized, controlled, single-blind clinical trial. Knee Surg. Sports Traumatol. Arthrosc..

[51] Grapar Žargi T., Drobnič M., Vauhnik R., Koder J., Kacin A. (2017). Factors predicting quadriceps femoris muscle atrophy during the first 12 weeks following anterior cruciate ligament reconstruction. Knee.

[52] Alavi A., Rezaeian N., Ganji R., Yaghoubi A. (2021). Effect of blood flow restriction on serum levels of some factors of muscle atrophy in male elite athletes after anterior cruciate ligament reconstruction. J. Basic Res. Med. Sci..

[53] Erickson L.N., Owen M.K., Casadonte K.R., Janatova T., Lucas K., Spencer K., Brightwell B.D., Graham M.C., White M.S., Thomas N.T. (2024). The Efficacy of Blood Flow Restriction Training to Improve Quadriceps Muscle Function After ACL Reconstruction. Med. Sci. Sports Exerc..

[54] Kaya Utlu D. (2023). Exercise. Functional Exercise Anatomy and Physiology for Physiotherapists.

[55] Whiteley R. (2019). Blood Flow Restriction Training in Rehabilitation: A Useful Adjunct or Lucy’s Latest Trick?. J. Orthop. Sports Phys. Ther..

[56] Wengle L., Migliorini F., Leroux T., Chahal J., Theodoropoulos J., Betsch M. (2022). The Effects of Blood Flow Restriction in Patients Undergoing Knee Surgery: A Systematic Review and Meta-analysis. Am. J. Sports Med..

[57] Charles D., White R., Reyes C., Palmer D. (2020). A systematic review of the effects of blood flow restriction training on quadriceps muscle atrophy and circumference post ACL reconstruction. Int. J. Sports Phys. Ther..

[58] Álvarez C.B., Santamaría P.I.-K., Fernández-Matías R., Pecos-Martín D., Achalandabaso-Ochoa A., Fernández-Carnero S., Martínez-Amat A., Gallego-Izquierdo T. (2021). Comparison of Blood Flow Restriction Training versus Non-Occlusive Training in Patients with Anterior Cruciate Ligament Reconstruction or Knee Osteoarthritis: A Systematic Review. J. Clin. Med..

[59] Christensen J.C., Goldfine L.R., Barker T., Collingridge D.S. (2015). What Can the First 2 Months Tell Us About Outcomes After Anterior Cruciate Ligament Reconstruction?. J. Athl. Train..

[60] Ithurburn M.P., Altenburger A.R., Thomas S., Hewett T.E., Paterno M.V., Schmitt L.C. (2018). Young athletes after ACL reconstruction with quadriceps strength asymmetry at the time of return-to-sport demonstrate decreased knee function 1 year later. Knee Surg. Sports Traumatol. Arthrosc..

[61] Neuman P., Owman H., Müller G., Englund M., Tiderius C.J., Dahlberg L.E. (2014). Knee cartilage assessment with MRI (dGEMRIC) and subjective knee function in ACL injured copers: A cohort study with a 20 year follow-up. Osteoarthr. Cartil..

[62] Ngurah G., Aryana W., Febyan F., Dimitri D., Limena S., Kuswara L.W. (2024). Functional Outcome of ACL Reconstruction Following Pre-reconstruction Rehabilitation vs. None Rehabilitation: A Systematic Review and Meta-analysis. Rev. Bras. Ortop..

[63] Giesche F., Niederer D., Banzer W., Vogt L. (2020). Evidence for the effects of prehabilitation before ACL-reconstruction on return to sport-related and self-reported knee function: A systematic review. PLoS ONE.

[64] Kacin A., Strazar K. (2011). Frequent low-load ischemic resistance exercise to failure enhances muscle oxygen delivery and endurance capacity. Scand. J. Med. Sci. Sports.

[65] Maga M., Wachsmann-Maga A., Batko K., Włodarczyk A., Kłapacz P., Krężel J., Szopa N., Sliwka A. (2023). Impact of Blood-Flow-Restricted Training on Arterial Functions and Angiogenesis—A Systematic Review with Meta-Analysis. Biomedicines.

[66] Wang X., Wang Y., Yang X., Nasiruddin N.J.B.M., Dong D., Bin Samsudin S., Qin X.-M. (2023). Effects of blood flow restriction training on bone metabolism: A systematic review and meta-analysis. Front. Physiol..

[67] Song Y., Wang H., Chen L., Shangguan Y., Jia H. (2023). Effects of blood flow restriction training on bone turnover markers, microstructure, and biomechanics in rats. Front. Endocrinol..

[68] Lixandrão M.E., Ugrinowitsch C., Berton R., Vechin F.C., Conceição M.S., Damas F., Libardi C.A., Roschel H. (2018). Magnitude of Muscle Strength and Mass Adaptations Between High-Load Resistance Training Versus Low-Load Resistance Training Associated with Blood-Flow Restriction: A Systematic Review and Meta-Analysis. Sports Med..

[69] Hughes L., Patterson S.D. (2019). Low intensity blood flow restriction exercise: Rationale for a hypoalgesia effect. Med. Hypotheses.

[70] Song J.S., Spitz R.W., Yamada Y., Bell Z.W., Wong V., Abe T., Loenneke J.P. (2021). Exercise-induced hypoalgesia and pain reduction following blood flow restriction: A brief review. Phys. Ther. Sport.

[71] Walker A., Hing W., Lorimer A. (2020). The Influence, Barriers to and Facilitators of Anterior Cruciate Ligament Rehabilitation Adherence and Participation: A Scoping Review. Sports Med. Open.

[72] Uchino S., Saito H., Okura K., Kitagawa T., Sato S. (2022). Effectiveness of a supervised rehabilitation compared with a home-based rehabilitation following anterior cruciate ligament reconstruction: A systematic review and meta-analysis. Phys. Ther. Sport.

[73] Gamble A.R., Pappas E., O’Keeffe M., Ferreira G., Maher C.G., Zadro J.R. (2021). Intensive supervised rehabilitation versus less supervised rehabilitation following anterior cruciate ligament reconstruction? A systematic review and meta-analysis. J. Sci. Med. Sport.

[74] Lundberg M., Archer K.R., Larsson C., Rydwik E. (2019). Prehabilitation: The Emperor’s New Clothes or a New Arena for Physical Therapists?. Phys. Ther..

[75] Smith T.O., Davies L., Hing C.B. (2010). Early versus delayed surgery for anterior cruciate ligament reconstruction: A systematic review and meta-analysis. Knee Surg. Sports Traumatol. Arthrosc..

[76] Reijman M., Eggerding V., van Es E., van Arkel E., Brand I.v.D., van Linge J., Zijl J., Waarsing E., Bierma-Zeinstra S., Meuffels D. (2021). Early surgical reconstruction versus rehabilitation with elective delayed reconstruction for patients with anterior cruciate ligament rupture: COMPARE randomised controlled trial. Br. Med. J..

[77] Wilk K.E., Arrigo C.A., Bagwell M.S., Finck A.N. (2021). Considerations with Open Kinetic Chain Knee Extension Exercise Following ACL Reconstruction. Int. J. Sports Phys. Ther..

[78] Pamboris G.M., Pavlou K., Paraskevopoulos E., Mohagheghi A.A. (2024). Effect of open vs. closed kinetic chain exercises in ACL rehabilitation on knee joint pain, laxity, extensor muscles strength, and function: A systematic review with meta-analysis. Front. Sports Act. Living.

[79] Perriman A., Leahy E., Semciw A.I. (2018). The effect of open-versus closed-kinetic-chain exercises on anterior tibial laxity, strength, and function following anterior cruciate ligament reconstruction: A systematic review and meta-analysis. J. Orthop. Sports Phys. Ther..

[80] Subirats Bayego E., Subirats Vila G., Soteras Martínez I. (2011). Exercise prescription: Indications, dosage and side effects. Med. Clin..

[81] Hansford H.J., Wewege M.A., Cashin A.G., Hagstrom A.D., Clifford B.K., McAuley J.H., Jones M.D. (2022). If exercise is medicine, why don’t we know the dose? An overview of systematic reviews assessing reporting quality of exercise interventions in health and disease. Br. J. Sports Med..

[82] Carter H.M., Littlewood C., Webster K.E., Smith B.E. (2020). The effectiveness of preoperative rehabilitation programmes on postoperative outcomes following anterior cruciate ligament (ACL) reconstruction: A systematic review. BMC Musculoskelet. Disord..

[83] Refalo M.C., Helms E.R., Hamilton D.L., Fyfe J.J. (2023). Influence of Resistance Training Proximity-to-Failure, Determined by Repetitions-in-Reserve, on Neuromuscular Fatigue in Resistance-Trained Males and Females. Sports Med. Open.

[84] Grgic J., Schoenfeld B.J., Orazem J., Sabol F. (2022). Effects of resistance training performed to repetition failure or non-failure on muscular strength and hypertrophy: A systematic review and meta-analysis. J. Sport Health Sci..

[85] Sieljacks P., Degn R., Hollaender K., Wernbom M., Vissing K. (2019). Non-failure blood flow restricted exercise induces similar muscle adaptations and less discomfort than failure protocols. Scand. J. Med. Sci. Sports.

[86] Husmann F., Mittlmeier T., Bruhn S., Zschorlich V., Behrens M. (2018). Impact of Blood Flow Restriction Exercise on Muscle Fatigue Development and Recovery. Med. Sci. Sports Exerc..

[87] Freitas E.D.S., Miller R.M., Heishman A.D., Aniceto R.R., Silva J.G.C., Bemben M.G. (2019). Perceptual responses to continuous versus intermittent blood flow restriction exercise: A randomized controlled trial. Physiol. Behav..

[88] Yasuda T., Loenneke J., Ogasawara R., Abe T. (2013). Influence of continuous or intermittent blood flow restriction on muscle activation during low-intensity multiple sets of resistance exercise. Acta Physiol. Hung..

[89] Caetano D., Oliveira C., Correia C., Barbosa P., Montes A., Carvalho P. (2021). Rehabilitation outcomes and parameters of blood flow restriction training in ACL injury: A scoping review. Phys. Ther. Sport.

[90] McEwen J.A., Owens J.G., Jeyasurya J. (2019). Why Is It Crucial to Use Personalized Occlusion Pressures in Blood Flow Restriction (BFR) Rehabilitation?. J. Med. Biol. Eng..

[91] Jacobs E., Rolnick N., Wezenbeek E., Stroobant L., Capelleman R., Arnout N., Witvrouw E., Schuermans J. (2023). Investigating the autoregulation of applied blood flow restriction training pressures in healthy, physically active adults: An intervention study evaluating acute training responses and safety. Br. J. Sports Med..

[92] Bond C.W., Hackney K.J., Brown S.L., Noonan B.C. (2018). Blood Flow Restriction Resistance Exercise as a Rehabilitation Modality Following Orthopaedic Surgery: A Review of Venous Thromboembolism Risk. JOSPT.

