# Supplementary material for: Blood Flow Restriction Training Prior to and After Anterior Cruciate Ligament Reconstruction: A Scoping Review
Source: J Funct Morphol Kinesiol. 2025 Nov 19;10(4):450. doi: 10.3390/jfmk10040450 (PMC12641707; doi:10.3390/jfmk10040450)
Supplement: Supplementary file 1 [file jfmk-10-00450-s001.zip › PRISMA CHECK-LIST (BFRT and ACLR).pdf]

PRISMA 2020 Checklist

Section and Topic	Item #	Checklist item	Location where item is reported
**TITLE**			
Title	1	Identify the report as a systematic review.	Blood Flow Restriction Training Prior to and After Anterior Cruciate Ligament Reconstruction: A Scoping Review**. (Page #1)**
**ABSTRACT**			
Abstract	2	See the PRISMA 2020 for Abstracts checklist.	Include all sections indicated in the JOSPT guide and use the following titles for each section: Study Design, Scoping Review, Objective, Literature Search, Study Selection Criteria, Data Synthesis, Results, Conclusion, Keywords. **(Page #1)**
**INTRODUCTION**			
Rationale	3	Describe the rationale for the review in the context of existing knowledge.	Our scoping review addresses the need to consolidate evidence on the effectiveness of BFR training in ACLR rehabilitation, a growing area of interest. Given the exploratory nature of our objectives, the scoping review approach is ideal for mapping existing literature, identifying key concepts, and highlighting gaps, thereby informing clinical practice and guiding future research. **(Page #2)**
Objectives	4	Provide an explicit statement of the objective(s) or question(s) the review addresses.	Our scoping review addresses the effectiveness of BFR-RT compared to non-BFR training in ACLR patients, focusing on preoperative and postoperative phases. These objectives aim to assess BFR's impact on body composition, neuromuscular responses and adaptations, self-reported questionnaires, functional measures, muscle blood flow, and return to activity. **(Page #2)**
**METHODS**			
Eligibility criteria	5	Specify the inclusion and exclusion criteria for the review and how studies were grouped for the syntheses.	The PICOS approach guided the selection of eligible sources, requiring articles to be peer-reviewed original research, available in full-text format, and published in Spanish, Portuguese, or English. **(Page #3,4)**
Information sources	6	Specify all databases, registers, websites, organisations, reference lists and other sources searched or consulted to identify studies. Specify the date when each source was last searched or consulted.	The systematic literature search utilized multiple databases including Web of Science,

Section and Topic	Item #	Checklist item	Location where item is reported
			PEDro, Scopus, PUBMED (MEDLINE), SportDiscus, and the Cochrane Library, covering from inception until April 1, 2024. Authors were also contacted for supplementary materials when necessary. **(Page #5 and Appendix A)**
Search strategy	7	Present the full search strategies for all databases, registers and websites, including any filters and limits used.	The complete electronic search strategy, meticulously documented, covers multiple databases including Web of Science, PEDro, Scopus, PubMed, SPORTDiscus, and Cochrane. Specific search terms such as "anterior cruciate ligament reconstruction" and "blood flow restriction" were utilized across relevant fields like title, abstract, and MeSH terms where applicable. This structured approach ensures thoroughness and replicability in gathering pertinent literature across diverse scholarly databases for the scoping review on ACLR and BFR. **(Page #5 and Appendix A)**
Selection process	8	Specify the methods used to decide whether a study met the inclusion criteria of the review, including how many reviewers screened each record and each report retrieved, whether they worked independently, and if applicable, details of automation tools used in the process.	The source selection process was conducted by two independent reviewers who screened search results based on predefined inclusion and exclusion criteria. In cases where discrepancies arose, a third evaluator resolved conflicts through consensus. This rigorous approach ensured that the final selection of sources for the scoping review was comprehensive and methodologically sound, aligning with established research objectives and criteria. **(Page #5 and Table 1)**
Data collection process	9	Specify the methods used to collect data from reports, including how many reviewers collected data from each report, whether they worked independently, any processes for obtaining or confirming data from study investigators, and if applicable, details of automation tools used in the process.	For the initial exploratory search, SWIFT-Reviewer was utilized. The selection and screening process was conducted using Rayyan, with two independent reviewers screening titles and abstracts on a blinded platform. Articles passing this stage were tracked using Citation Chaser for backward and forward citations. General characteristics of included studies were recorded in one table. Another table detailed exercise training doses

Section and Topic	Item #	Checklist item	Location where item is reported
			and BFR parameters used in the selected studies. Study variables were categorized into sections. **(Page #5)**
Data items	10a	List and define all outcomes for which data were sought. Specify whether all results that were compatible with each outcome domain in each study were sought (e.g. for all measures, time points, analyses), and if not, the methods used to decide which results to collect.	The variables for which data were sought include body composition, neuromuscular adaptations and responses, functional measurements, self-reported questionnaires, muscle blood flow, and return to activity time. Body composition variables encompassed muscle volume, thickness, cross-sectional area, site-specific bone mass, bone mineral density, whole limb lean mass, and biopsy analysis. Neuromuscular adaptations and responses included maximum voluntary isometric contraction, isokinetic strength, activation of the vastus medialis, central activation ratio of knee extensors, and fatigue indices. Functional measurements covered various tests and values related to functionality. Self-reported questionnaires included patient-reported scales and questionnaires. Muscle blood flow was measured directly, and return to activity time was documented. Data were sought for all measures, time points, and analyses compatible with each outcome domain in each study. **(Page #5)**
	10b	List and define all other variables for which data were sought (e.g. participant and intervention characteristics, funding sources). Describe any assumptions made about any missing or unclear information.	Other variables for which data were sought included participant characteristics (age, sex, baseline fitness level), intervention details (type, duration, frequency, intensity, and progression of training), and funding sources. Additionally, we recorded study features (author, study design), exercise training prescription (FITT-VP: Frequency, Intensity, Time, Type, Volume, and Progression), cuff parameters (device and pressure), and outcome measures (within and between group changes). **(Page #5)**
Study risk of bias assessment	11	Specify the methods used to assess risk of bias in the included studies, including details of the tool(s) used, how many reviewers assessed each study and whether they worked independently, and if applicable, details of automation tools used in the process.	The methodological quality of the selected RCTs and QRCTs was assessed using the PEDro scale. Additionally, the CERT scale

Section and Topic	Item #	Checklist item	Location where item is reported
			was used to review the reporting of training parameters and BFR protocols in the included studies. Risk of bias assessment was facilitated using SWIFT-Reviewer, with two independent reviewers evaluating each study's risk of bias. They worked independently to ensure objectivity. **(Page #5 and Table 1)**
Effect measures	12	Specify for each outcome the effect measure(s) (e.g. risk ratio, mean difference) used in the synthesis or presentation of results.	For each outcome, the effect measures used in the synthesis or presentation of results included mean differences and percentages of change, whenever possible. In Table 3**,** we present the exercise training doses and BFR parameters used in the selected studies, along with the percentages of change, statistically significant interactions, and the corresponding p-values. **(Page #5 and Table 3)**
Synthesis methods	13a	Describe the processes used to decide which studies were eligible for each synthesis (e.g. tabulating the study intervention characteristics and comparing against the planned groups for each synthesis (item #5)).	To determine the eligibility of studies for each synthesis, we first defined clear inclusion and exclusion criteria based on intervention type, population, study design, and outcome measures. TABLE 2 facilitated the tabulation of study interventions and general characteristics, including moment of intervention, author, study design, autograft type, intervention time, population, sample characteristics, groups, and studied variables. We compared these characteristics against the planned groups for each synthesis. Studies that met the criteria and had relevant data for the planned groups were included in the synthesis. **(Page #5 and Table 2)**
	13b	Describe any methods required to prepare the data for presentation or synthesis, such as handling of missing summary statistics, or data conversions.	To prepare the data for presentation and synthesis, we addressed missing summary statistics by contacting study authors when possible. No data imputation or transformation was performed. Data were standardized to ensure consistency across studies without any additional adjustments. **(Page #5)**
	13c	Describe any methods used to tabulate or visually display results of individual studies and syntheses.	We used tables to summarize study characteristics, interventions, and outcomes. The PRISMA diagram was employed to

Section and Topic	Item #	Checklist item	Location where item is reported
			illustrate the selection process of studies. Additionally, the PEDro scale was presented in a summary figure and as Table percentage breakdown by item in its own graphic. The CERT scale was also displayed in tables, with percentage breakdowns provided for clarity. **(Page #5)**
	13d	Describe any methods used to synthesize results and provide a rationale for the choice(s). If meta-analysis was performed, describe the model(s), method(s) to identify the presence and extent of statistical heterogeneity, and software package(s) used.	In the scoping review, narrative synthesis was used due to methodological variability. A meta-analysis was not feasible. Tables summarized study characteristics, and descriptive statistics provided an overview of observed effects. **(Page #5)**
	13e	Describe any methods used to explore possible causes of heterogeneity among study results (e.g. subgroup analysis, meta-regression).	Descriptive analysis and comparative tables were used to explore possible causes of heterogeneity among study results. **(Page #5)**
	13f	Describe any sensitivity analyses conducted to assess robustness of the synthesized results.	No direct sensitivity analyses were conducted due to the lack of meta-analysis. However, the use of quality assessment tools like PEDro and CERT supported the robustness of the findings. **(Page #5)**
Reporting bias assessment	14	Describe any methods used to assess risk of bias due to missing results in a synthesis (arising from reporting biases).	To assess risk of bias due to missing results (reporting biases), SWIFT-Reviewer and citation chasing were used to search for unpublished studies or gray literature. These methods helped ensure a comprehensive review and better understanding of the study topic. **(Page #5)**
Certainty assessment	15	Describe any methods used to assess certainty (or confidence) in the body of evidence for an outcome.	To assess the certainty of the body of evidence, the PEDro scale and CERT were used. **(Page #5)**
**RESULTS**			
Study selection	16a	Describe the results of the search and selection process, from the number of records identified in the search to the number of studies included in the review, ideally using a flow diagram.	**(Page #6 and Figure 1)**
	16b	Cite studies that might appear to meet the inclusion criteria, but which were excluded, and explain why they were excluded.	**(Page #6 and Figure 1)**
Study characteristics	17	Cite each included study and present its characteristics.	**(Page #6-8, Figure 2 and Table 2)**

PRISMA 2020 Checklist

Section and Topic	Item #	Checklist item	Location where item is reported
Risk of bias in studies	18	Present assessments of risk of bias for each included study.	**(Page #6-8 and Figure 2 and APPENDIX B and C).**
Results of individual studies	19	For all outcomes, present, for each study: (a) summary statistics for each group (where appropriate) and (b) an effect estimate and its precision (e.g. confidence/credible interval), ideally using structured tables or plots.	For all outcomes, summary statistics for each group and effect estimates with their precision (e.g., confidence intervals) were presented, using structured tables or plots whenever possible. All significant results were shown. **(Page #6-8, 14 and Table 2,3)**
Results of syntheses	20a	For each synthesis, briefly summarise the characteristics and risk of bias among contributing studies.	**(Page #8 and Table 2)**
	20b	Present results of all statistical syntheses conducted. If meta-analysis was done, present for each the summary estimate and its precision (e.g. confidence/credible interval) and measures of statistical heterogeneity. If comparing groups, describe the direction of the effect.	**(Page #39-41 and Table 3)**
	20c	Present results of all investigations of possible causes of heterogeneity among study results.	**(Page #39-41)**
	20d	Present results of all sensitivity analyses conducted to assess the robustness of the synthesized results.	**(Page #39-41 and Table 3)**
Reporting biases	21	Present assessments of risk of bias due to missing results (arising from reporting biases) for each synthesis assessed.	**N/A**
Certainty of evidence	22	Present assessments of certainty (or confidence) in the body of evidence for each outcome assessed.	**(Page #14 and Appendix B)**
**DISCUSSION**			
Discussion	23a	Provide a general interpretation of the results in the context of other evidence.	**(Page #41-44)**
	23b	Discuss any limitations of the evidence included in the review.	**(Page #44-45)**
	23c	Discuss any limitations of the review processes used.	**(Page #44-45)**
	23d	Discuss implications of the results for practice, policy, and future research.	**(Page #41,44 and 46)**
**OTHER INFORMATION**			
Registration and protocol	24a	Provide registration information for the review, including register name and registration number, or state that the review was not registered.	**(Page #3)**
	24b	Indicate where the review protocol can be accessed, or state that a protocol was not prepared.	**(Page #3)**
	24c	Describe and explain any amendments to information provided at registration or in the protocol.	**N/A**
Support	25	Describe sources of financial or non-financial support for the review, and the role of the funders or sponsors in the review.	**(Page #46)**
Competing interests	26	Declare any competing interests of review authors.	**(Page #46)**
Availability of data, code and other materials	27	Report which of the following are publicly available and where they can be found: template data collection forms; data extracted from included studies; data used for all analyses; analytic code; any other materials used in the review.	**N/A**

From: Page MJ, McKenzie JE, Bossuyt PM, Boutron I, Hoffmann TC, Mulrow CD, et al. The PRISMA 2020 statement: an updated guideline for reporting systematic reviews. BMJ 2021;372:n71. doi: 10.1136/bmj.n71
For more information, visit: http://www.prisma-statement.org/
